# Eukaryotic G protein-coupled receptors as descendants of prokaryotic sodium-translocating rhodopsins

**DOI:** 10.1186/s13062-015-0091-4

**Published:** 2015-10-15

**Authors:** Daria N. Shalaeva, Michael Y. Galperin, Armen Y. Mulkidjanian

**Affiliations:** School of Physics, Osnabrueck University, 49069 Osnabrueck, Germany; School of Bioengineering and Bioinformatics, Lomonosov Moscow State University, Moscow, 119992 Russia; A.N. Belozersky Institute of Physico-Chemical Biology, Lomonosov Moscow State University, Moscow, 119992 Russia; National Center for Biotechnology Information, National Library of Medicine, National Institutes of Health, Bethesda, MD 20894 USA

**Keywords:** Vision, Bacteriorhodopsin, Halorhodopsin, Sensory rhodopsin, Proteorhodopsin, Opioid receptor, GPCR, Evolution, Signal transduction, Chemoreceptor

## Abstract

**Abstract:**

Microbial rhodopsins and G-protein coupled receptors (GPCRs, which include animal rhodopsins) are two distinct (super) families of heptahelical (7TM) membrane proteins that share obvious structural similarities but no significant sequence similarity. Comparison of the recently solved high-resolution structures of the sodium-translocating bacterial rhodopsin and various Na^+^-binding GPCRs revealed striking similarity of their sodium-binding sites. This similarity allowed us to construct a structure-guided sequence alignment for the two (super)families, which highlighted their evolutionary relatedness. Our analysis supports a common underlying molecular mechanism for both families that involves a highly conserved aromatic residue playing a pivotal role in rotation of the 6th transmembrane helix.

**Reviewers:**

This article was reviewed by Oded Beja, G. P. S. Raghava and L. Aravind.

**Electronic supplementary material:**

The online version of this article (doi:10.1186/s13062-015-0091-4) contains supplementary material, which is available to authorized users.

## Findings

The evolutionary relation between two large groups of sensory membrane proteins, namely the G-protein coupled receptors (GPCRs) and microbial rhodopsins (MRs) has been puzzling biologists for almost four decades. Both (super)families contain integral membrane proteins that consist of 7 transmembrane (TM) helices surrounding a relatively polar core [1–3]. In most of the studied GPCRs, binding of the sensed ligand molecule (agonist) causes a conformational change in the helical bundle that promotes an interaction with a GTP/GDP binding protein, which then triggers the intracellular signal cascade [4, 5]. The GPCR (super)family also includes retinal-containing visual rhodopsins, which are used by animals to sense light [2, 6–9]. The GPCRs are divided into several families, the major of which are rhodopsin-like receptors (class A), secretin receptors (class B), glutamate receptors (class C), fungal mating pheromone receptors (class D), cAMP receptors (class E), and frizzled receptors (class F) [10]. These receptors, widespread among eukaryotes, are being intensively studied for their ability to regulate various cellular processes. Human GPCRs serve as targets for numerous drugs, see [4, 11, 12] for reviews. The rhodopsin-like receptors (Class A GPCRs) make the largest GPCR family with more than 700 representatives encoded in the human genome [13].

Microbial rhodopsins (also known as type I rhodopsins) are retinal-containing membrane proteins that function either as light-driven ion pumps or as light sensors in many bacteria and archaea, as well as in some primitive eukaryotes [2, 6, 14–17]. MRs represent a distinct family within a large group of biochemically poorly characterized bacterial 7TM membrane receptors [18, 19] and differ from other heptahelical receptors in their ability to bind retinal.

The overall similarity of heptahelical bundles, as well as similar roles as photoreceptors has long prompted suggestions on the evolutionary relationship between visual rhodopsin, a GPCR, and MRs [1, 3, 17, 20], as well as, more generally, on the evolutionary relatedness of GPCRs and MRs [18, 20–22]. However, the attempts to find a significant sequence similarity between the two types of rhodopsins or just trace the conservation of retinal-binding residues brought no conclusive results [1, 23, 24]. Even the availability of the 3D structures of the visual rhodopsin and other GPCRs [7, 8, 25–28] and of several MRs [2, 16, 29–31] did not clarify the evolutionary relationship of the two protein families. Accordingly, to distinguish the seven TM helices of MRs and GPCRs, they are routinely referred to as helices A-F and 1–7, respectively.

The comparison of MRs and GPCRs has been additionally complicated by the uncertainty on whether all eukaryotic 7TM-receptors, including GPCRs, have monophyletic origin [10, 18, 19, 32, 33]. It has been shown that some groups of eukaryotic 7TM-receptors operate in a G-protein-independent way; they were dubbed GPCR-like proteins [19, 34]. Specifically, there is no statistically significant sequence similarity between the glutamate receptors (class C GPCRs) and other GPCRs. Phylogenetic analyses traced the glutamate receptors and cAMP receptors (class E GPCRs) to the eukaryotic root, and the latter family has been proposed to be the ancestor of the GPCRs of classes A, B, D, and F [32, 33]. The recent resolution of the first two structures of glutamate receptors, however, has revealed their overall structural similarity to other classes of GPCRs [35, 36], supporting the common origin of all GPCR-like proteins.

The discovery of the sodium-translocating microbial rhodopsins (NRs) [16, 37–39] and the recent characterization of the 3D structures of a representative Na^+^-transporting rhodopsin from the bacterium *Krokinobacter eikastus* (KR2) with a resolution of 1.45 Å [40] and 2.3 Å [41] prompted us to reinvestigate the long-standing conundrum on the evolutionary relation between GPCRs and MRs. We report here that crystal structures of the Na^+^-transporting rhodopsin provide the missing piece of the puzzle and support the relationship between the two (super)families of 7TM proteins by revealing a deep, sodium-based link between the MRs and GPCRs.

Indeed, sodium ions have long been known to affect binding of agonists in many class A GPCRs [27]; the binding of an extracellular Na^+^ ion in the middle of the 7TM bundle of several such GPCRs has been recently characterized in detail [25–28, 42]. Given that the ability to bind Na^+^ ions is shared between the Na^+^-transporting rhodopsin and some GPCRs, we have undertaken a structural comparison of these two protein families.

Previously, we have compared the Na^+^-binding sites of bacterial and archaeal Na^+^-translocating ATP synthases while reconstructing their evolutionary history. Rotary ATP synthases produce ATP at the expense of transmembrane difference in the electrochemical potential of protons (in the vast majority of organisms, including plants and animals) or sodium ions (in some anaerobic prokaryotes). From the comparison of the Na^+^-binding sites, the ancient state of this enzyme could be reconstructed as a Na^+^-exporting, ATP-driven pump, one of the ancient sodium export pumps that could keep the [K^+^]/[Na^+^] ratio in the cell cytoplasm over unity [43–46]. Since many key cellular systems, traceable to the Last Universal Cellular Ancestor (LUCA) and including the protein synthesis, are activated by K^+^ ions and inhibited by Na^+^ ions, even the primordial cells should have had systems for Na^+^ export [46–48]. Our phylogenomic analysis even suggested that a particular family of rotary ATPases, that we dubbed N-ATPases, contains enzymes that still operate as Na^+^ export pumps in modern organisms [49]. This prediction has been experimentally confirmed for a cyanobacterial N-ATPase [50].

**Fig. 1:**
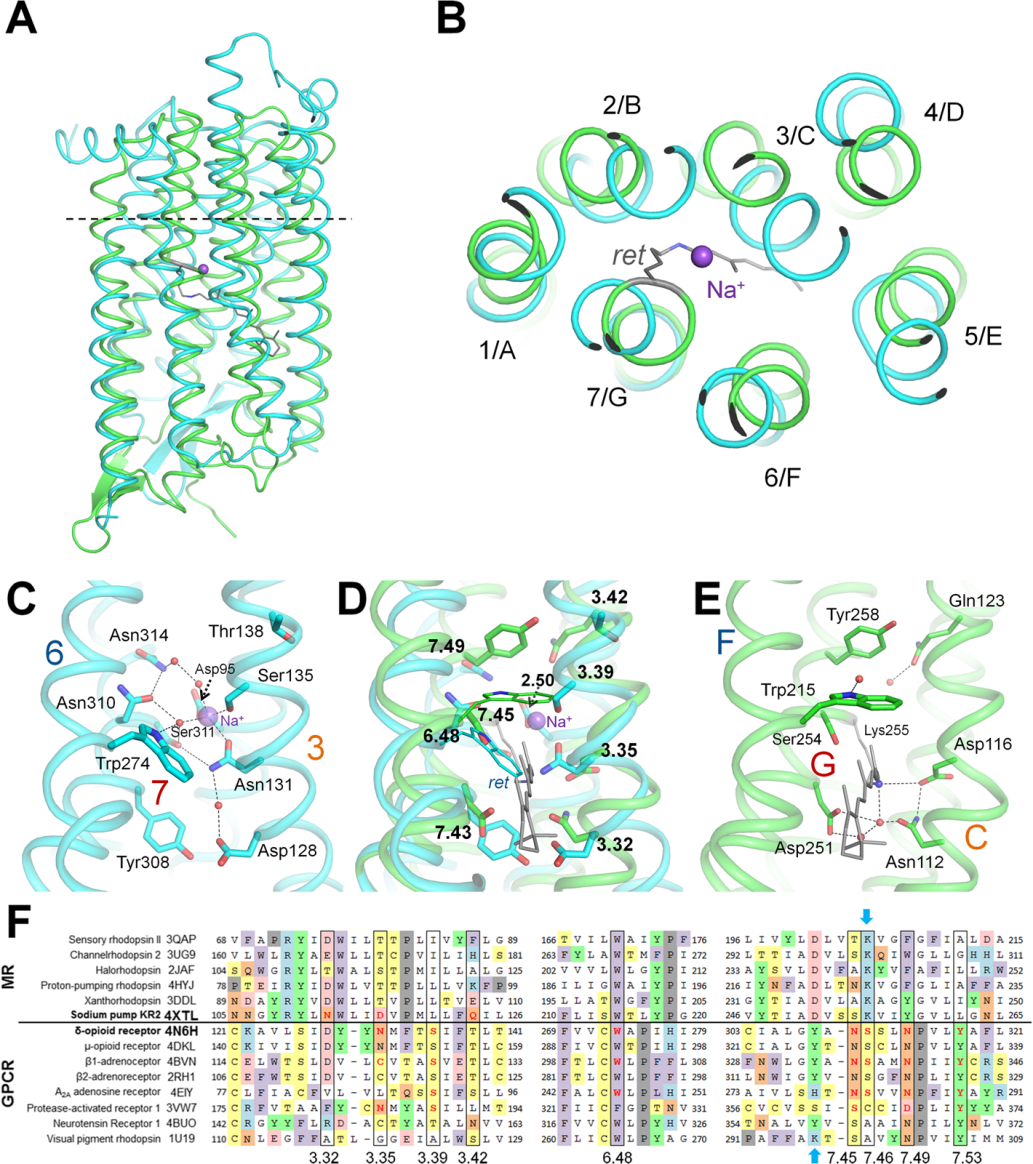
Structure-guided alignment of GPCRs and MRs. **a**, **b**, structural superposition of the entire structures of the sodium-translocating microbial rhodopsin KR2 (PDB: 4XTL, green) and the Na^+^-bound δ-opioid receptor δ-OR (PDB: 4N6H, blue), constructed using the PDBeFold tool [53]; the RMSD is 3.8 Å over 189 aligned residues with 14 % identity. The Na^+^ ion in the δ-OR structure is shown as a pink sphere. The retinal molecule bound to KR2 lysine residue is shown in grey. **a**, side view; **b**, top view from the extracellular side, the structures were cut along the dashed line. **c**-**e**, Na^+^-binding sites of the Na^+^-bound δ-opioid receptor δ-OR (panel **c**, PDB: 4N6H, cyan) [26], the sodium-translocating microbial rhodopsin KR2 (panel E, PDB 4XTL, green, the helices of KR2, as in other MRs, are denoted by letters from A to G) [40], and their superposition (panel **d**). Residue numbers are according to the Ballesteros–Weinstein nomenclature [56, 57]. Residues involved in coordination of Na^+^ ions in 4N6H (panel **c**) and 4XTL (panel **e**) are shown as sticks; the Na^+^ ion (panels **c**, **d**) and the imino group of the Schiff base (panels **d**, **e**) are shown as pink and blue spheres, respectively; water molecules are shown as small red spheres. The retinal molecule in panels **d** and **e** is shown in grey. For the visualization purposes only, we used the PDBeFold algorithm [53] to construct a superposition of KR2 and δ-OR with helices 4 and 5 removed. The resulting superposition provided a better overlap in the Na^+^-binding area with a local RMSD of 2.97 Å over 135 residues. F, structure-guided multiple sequence alignment of helices 3/C, 6/F, and 7/G of MRs and GPCRs. PDB: 3QAP, sensory rhodopsin II [93]; PDB: 3UG9, channelrhodopsin [94]; PDB: 2JAF, halorhodopsin [95]; PDB: 4HYJ, proton-pumping bacteriorhodopsin, [96]; PDB: 3DDL, xanthorhodopsin [30]; PDB: 4XTL, sodium pumping rhodopsin (KR2) [40]; PDB: 4N6H, δ-opioid receptor (δ-OR) [26]; PDB: 4DKL, μ-opioid receptor [97]; PDB: 4BVN, β1-adrenoceptor [28]; PDB: 2RH1, β2-adrenoreceptor [98]; PDB 4EIY, A(2A) adenosine receptor [42]; PDB: 3VW7, protease-activated receptor 1 [25]; PDB: 4BUO, neurotensin receptor 1 [99]; PDB: 1U19, visual pigment rhodopsin [100]. The boxes indicate positions corresponding to the known Na^+^-binding residues in GPCRs (see also Additional file 1: Figure S1, S4 for a complete structure-based sequence alignment and Additional file 1: Figure S5 for a multiple structural superposition). The residues that are involved in Na^+^ binding, as inferred from structural or mutation data [16, 25–28, 38, 40, 41, 51] are colored red. The retinal binding Lys residues of bovine eye rhodopsin and MRs are indicated by blue arrows. Aromatic amino acids are shaded violet, proline is shaded gray, tyrosine is shaded green, other residues capable of forming hydrogen bonds are shaded by different colors depending on their electric charge

Encouraged by these results, and searching for other vestiges of the primordial “Sodium World”, we used here a similar approach to perform a comparative analysis of the Na^+^-binding sites in KR2 (PDB: 4XTL) [40] and various GPCRs. We have started from a manual superposition of the likely Na^+^-binding ligands of KR2, identified in refs. [16, 38, 40, 41, 51], and the Na^+^-binding ligands of Na^+^-bound δ-opioid receptor (hereafter δ-OR, PDB: 4N6H) [26]. In both proteins, the 3^d^ and 7^th^ helices contribute the whole sets of potential Na^+^ ligands; upon manual superposition of the segments of two structures using PyMOL [52] these ligands overlapped (not shown). Next, we tried out several different sequence alignment and structural superposition software packages to align the entire proteins. The Na^+^ligands got aligned within completely superposed structures when we used the secondary structure matching (SSM) method implemented on the PDBeFold server [53] (http://www.ebi.ac.uk/msd-srv/ssm/). The alignment produced by PDBeFold had an RMSD of 3.8 Å with all seven helices (a total of 189 residues) aligned (Fig. 1a, b). We used this structural alignment as a basis for the sequence alignments shown in Fig. 1f and Additional file 1: Figure S1, S4, as well as for superposition of multiple structures in Additional file 1: Figure S5.

In the end, we have found that the list of structures similar to KR2 (PDB: 4XTL) [40] on the Protein Data Bank (PDB) web server [54], which uses the jFATCAT-rigid algorithm to deduce structural similarity [55], already includes, just after other MRs, structures of several Na^+^-dependent GPCRs with the similarities that are characterized by *P*-values of ~10^−8^ and structural alignments with RMSD of 4.1-4.5 Å (see Additional file 1: Table S1). Although these RMSD values were somewhat larger than those obtained using PDBeFOLD, the possibility of comparing these pre-aligned structures and the respective sequence alignments proved to be useful in searching for the similarities between different MRs and GPCRs. Specifically, for some KR2/GPCR pairs, the jFATCAT-rigid algorithm produced a distinct alignment with only six superposed helices, where the helices of the GPCR structures were shifted, by one helix turn relatively to KR2, towards the cytoplasmic side of the membrane, as compared to the superposition pattern shown in Fig. 1, see Additional file 1: Figures S2, S3 and the discussion below.

In Fig. 1c-e, those residues of KR2 that are expected to coordinate the Na^+^ ion during its light-triggered passage through the mid-plane of the membrane [16, 38, 40, 41, 51] overlap with the actual Na^+^ ligands of δ-OR in the 3^d^ and 7^th^ helices. In GPCRs, the respective residues have been shown to form the Na^+^-binding pocket not only in δ-OR (Fig. 1c), but also in the human protease-activated receptor 1 (PAR1) [25], human A_2A_ adenosine receptor [42], and in β_1_-adrenoreceptor [27, 28]. Specifically, Asp116 of the characteristic “NDQ” motif of Na^+^-transporting MRs [38] in the helix 3 of KR2 matched Asn131^3.35^ of δ-OR (the numbers in superscripts indicate GPCR helix and residue numbers according to the Ballesteros–Weinstein nomenclature [56, 57]), while Ser254 and Tyr258 of KR2 matched Asn310^7.45^ and Asn314^7.49^ of δ-OR (helix 7). In addition, Asn112 of the “NDQ” motif of KR2 matched Asp128^3.32^ of δ-OR (helix 3), whereas Asp251of KR2 matched Tyr308^7.43^ of δ-OR (helix 7). Matching of Asp and Asn residues in the alignments (Fig. 1, Additional file 1: Figure S4) is justified by the comparable potency of carboxyl and caroxamide groups to bind Na^+^ and K^+^ ions in proteins [13, 58], in a sharp contrast with the inability of carboxamide groups to bind protons.

In KR2, the imino group of the Schiff base that connects the retinal with Lys255 plugs the potential Na^+^-binding site when KR2 is in the ground state [39–41] (Fig. 1e). Functional and structural studies of the Na^+^ translocating rhodopsins [16, 38, 40, 41, 51, 59] indicate that photoisomerization of the retinal, by twisting the side chain of Lys255, is bound to unplug the Na^+^-binding site of KR2, cause deprotonation of the Schiff base (as it has been shown in bacteriorhodopsin [60]), and, concurrently, induce an outward movement of the 6^th^ helix (F-helix), opening a cleft that is needed for ion translocation across the hydrophobic part of the protein, as in other microbial rhodopsins [21, 61–65]. The nucleophilic nitrogen atom of the deprotonated Schiff base would thereby provide one more ligand for the Na^+^ ion on its way through KR2. The resulting layers of polar residues along the interacting surfaces of helices 3 and 7 (Fig. 1d) yield a typical cation-conducting structure, which was previously described in ion channels [66]. Binding of the Na^+^ ion by the stretches of polar residues of the 3^d^ and 7^th^ helices, which are conserved throughout MRs and GPCRs (see a structure-based sequence alignment in Fig. 1f, Additional file 1: Figure S1 and S4), strongly support a common origin of all these proteins.

According to the alignment on Fig. 1f (see also Additional file 1: Figures S4, S5), only a single residue of the 6^th^ helix (Trp215 in KR2 and Trp274^6.48^ in δ-OR) is conserved throughout MRs and GPCRs. In MRs, this residue couples photoisomerization of the retinal to the aforementioned major rotation of helix F (see Additional file 1: Figure S6), which leads either to ion translocation (e.g., in bacteriorhodopsin [21, 61, 62, 64], halorhodopsin [31], Na^+^-transocating rhodopsin [59] and channelrhodopsin [67, 68]) or to signal transduction (in sensory rhodopsins [2, 63]). In GPCRs, this Trp residue mediates signal transduction since it interacts with ligands (see Additional file 1: Figure S7), as well as, via a water molecule, with the Na^+^ ion in some Na^+^-binding GPCRs, see Fig. 1c [5, 25, 27]. The conformational change of this Trp residue in response either to the binding of substrate (see Additional file 1: Figure S7 and ref. [5]) or to the photoisomerization of retinal in rhodopsin [8, 9] triggers the rotation and tilting of helix 6. The broad conservation of this “pivotal” [8, 9] Trp residue likely reflects an already mentioned [21] underlying commonality in the molecular mechanisms in MRs and GPCRs. Most MRs and GPCRs appear to be relying on a forced reorientation of this bulky, hydrogen-bonded Trp residue, which results in the movement of the 6^th^ helix. The pivotal function of a residue in this position also holds true in the rare instances when this Trp residue is replaced, e.g. by Phe in the human PAR1 [25].

The structure-guided sequence alignment of δ-OR and KR2, which we have expanded by including additional sequences of structurally resolved MRs and GPCRs (Fig. 1f and Additional file 1: Figure S4), shows that KR2 represents an intermediate case between other MRs and class A GPCRs. This position might reflect its proximity to the common prokaryotic ancestor of both (super)families, which, apparently, contained a Na^+^-binding site and probably also was a light-driven sodium export pump (NR). While initially discovered in *Flavobacteria* [37, 38], NRs are widespread among bacteria (see Additional file 1: Figure S8), which is consistent with an ancient origin of these enzymes.

**Fig. 2:**
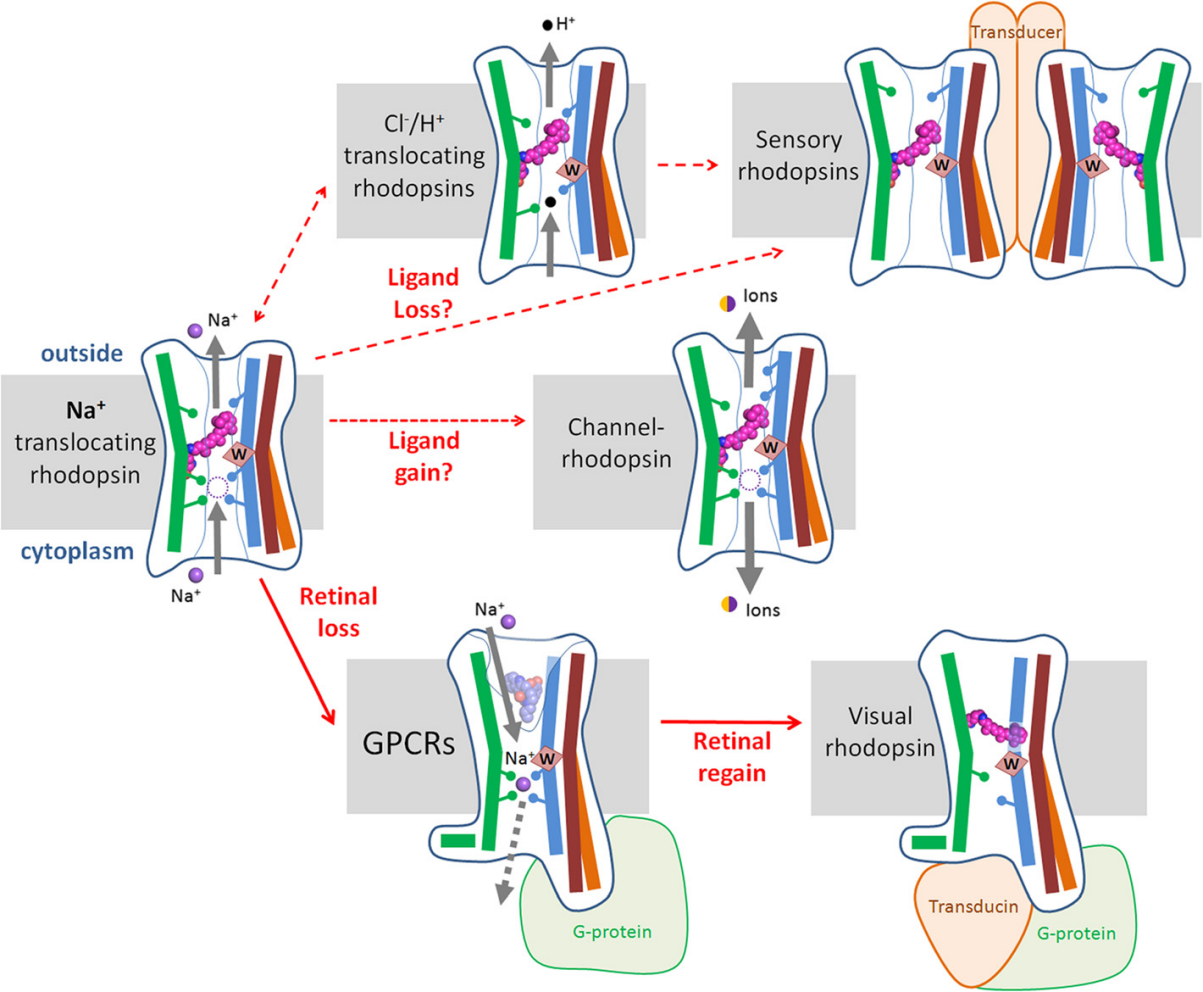
Proposed scheme of the evolution of MRs and GPCRs. Only three helices of MRs and GPCRs are depicted. Helix 3 (helix C of MRs) is colored blue, helix 6 (helix F of MRs) is colored brown in the “closed” conformation and orange in the “open” conformation, helix 7 (helix G of MRs) is colored green. The scheme illustrates the proposed order of appearance of functions in evolution as a series of gains and losses. The losses of retinal could lead to numerous bacterial 7TM receptors [18, 19] (not shown on the scheme) and to the Na^+^-binding precursor of most GPCRs. In the course of further evolution, many GPCRs seemingly lost the ability to bind Na^+^ (not shown on the scheme). The reacquisition of the retinal by a class A GPCR yielded a visual rhodopsin. See the text for further details

There is no general consensus on phylogenetics of either GPCRs [10, 32, 33] or MRs [39, 69], not to mention that MRs are prone to (virus-mediated) lateral gene transfer [70, 71]. Still, the conservation of certain structural traits in MRs and GPCRs allows us to formulate a likely scenario of the emergence of eukaryotic GPCRs from a light-driven sodium export pump, see Fig. 2. Pumping the Na^+^ ion across the membrane requires negatively charged/polar groups placed along the ion path that would compensate the positive charge of Na^+^ [72]. To guarantee one-way pumping, the system must also contain a switching mechanism [73]. In modern ion-pumping MRs, switching involves the conserved Trp residue of helix 6; the light-induced tilting of this helix opens a conduit for the translocated ions [21, 31, 59, 61, 62, 64, 67, 68]. The ability to turn/tilt the 6^th^ helix around the conserved Trp residue, which is shared by MRs and GPCRs, suggests that the ancestral form of the protein was already capable of doing that. Such a forced tilting of the α-helix in the middle of a TM segment, which is accompanied by large scale conformational changes [21, 31, 59, 61, 62, 64, 65, 67, 68], is an unusual feature that probably could have emerged just once, being shaped by the light-driven isomerization of the retinal.

Once this machinery for light-driven sodium translocation has emerged, it could evolve in different directions, giving rise to a variety of heptahelical proteins with different functions. As depicted in Fig. 2, the relations between these proteins can be described in terms of gains and losses of the residues involved in binding of Na^+^ and/or retinal. Proton- and chloride-translocating rhodopsins, as well as diverse sensory rhodopsins [16–19, 74–77], have fewer ion-binding ligands than NR, see Fig. 2 and *cf* with Fig. 1f, Additional file 1: Figure S4a and S10a. On the other hand, emergence of additional ionizable groups in the transmembrane helices could increase the ion conductivity ultimately yielding light-gated channels of channelrhodopsins [76, 78].

We have previously argued that the membranes of the first cells should have been tight to sodium ions, but not to protons [44–46]. Eukaryotes usually do not maintain high proton gradient at their cell membranes, with plasma membranes of many animal cells being even  leaky to protons [79]; therefore the early eukaryotes could particularly benefit from sodium-translocating rhodopsins. In such a rhodopsin, the retinal-binding ability could get lost, e.g. as a result of the loss of the retinal-binding lysine residue, as described in some MR lineages [80, 81]. The loss of the retinal would have left an empty void and a structurally compromised protein. Such a protein, however, could be re-stabilized by converting the transient Na^+^-binding site in the middle of the membrane into a permanent one. Nascent Na^+^-coordinating residues, such as Asp^2.50^, absent from MRs, could functionally replace the retinal moiety, see Fig. 2 and Additional file 1: Figure S1.

In the absence of retinal, the Trp residue of helix 6 would reach the sodium ion and provide an additional water-bridged coordination bond (Fig. 1c). Small organic ligands, by filling the cavity, would further stabilize the protein, paving the way to the emergence of specific Na^+^-binding 7TM receptors for small organic molecules, which, as we believe, were the ancestors of most modern GPCRs (see Fig. 2). In these proteins, the turning/twisting of the 6^th^ helix would be controlled by the interaction of ligands and/or Na^+^ ions with the key Trp residue.

The suggested emergence of most GPCRs from NRs is supported by the following observations:

1. The conversion of the transient Na^+^-binding site in the middle of the membrane into a permanent one by replacing Asp251 by Glu has been described for a mutant of a Na^+^-translocating bacterial rhodopsin of *Gillisia limnaea* [51].
2. The Na^+^ ions have been shown to stabilize the GPCRs in an inactive state (antagonist-bound or ligand-free) [27, 28]. In contrast, the structures of agonist-bound, active GPCRs show no space for a Na^+^ ion, which has already prompted a suggestion that activation of these GPCRs might be coupled with the release of the sodium ion to the opposite, cytoplasmic side of the membrane [27, 82]. It has been argued that Na^+^ transfer, promoted by the transmembrane sodium gradient, could provide an energy source, assisting GPCR signaling by small molecules [27].
3. The recently described GPCR-like, 7TM plant receptor, *Arabidopsis* protein GCR1 (At1g48270, UniProt: O04714), contains a set of Na^+^ ligands and has been suggested to bind Na^+^ [34, 83]. The protein shows sequence similarity to class A GPCRs [34] and has close homologs in the genomes of Amoebozoa, Ciliates and Choanoflagellates (see Additional file 1: Figure S9). These GCR1 homologs, some of which have been earlier categorized as class E/cAMP GPCRs [10, 32, 84] and also proven to be receptors [85], contain most of the Na^+^ ligands and the conserved Trp residue in the 6^th^ helix (Additional file 1: Figure S9). It has been already suggested that class E GPCR-like proteins most closely resemble the common ancestor of all GPCRs, except glutamate receptors (class C GPCRs) [32, 33]. It seems likely that this common ancestor of most GPCRs contained a Na^+^ binding site. The ability to bind Na^+^, apparently, has been retained by GPCRs of class A [25–28] and, probably, of class E (cAMP receptors), see [34] and Additional file 1: Figure S9, but could have been lost in other classes of GPCRs, as it also happened in the majority of rotary ATPases [44].

The loss of the retinal moiety could also cause certain rearrangement of the protein. As mentioned above, in case of some KR2/GPCR pairs, the jFATCAT-rigid algorithm yielded superposition pattern that differed from those shown in Fig. 1 in that all the helices of the GPCR were shifted by one turn relative to the KR2 and only six TM helices of seven overlapped (cf. Additional file 1: Figures S2 and S3). It is noteworthy that the *P*-values of such alternative, “incomplete” structural alignments were comparable with those of the alignments with seven overlapping helices, as obtained by the same algorithm for other KR2/GPCR pairs (see the captions to Additional file 1: Figures S2 and S3). This could be due to the presence of superimposed segment(s) with particularly high local score(s) in the alternative alignments. We have noted that the putative Na^+^ ligands matched better in case of alternative alignments of helix G of MRs with helix 7 of GPCRs (Additional file 1: Figure S10a) than in the “standard” alignment (Fig. 1 and Additional file 1: Figure S4). In contrast, the alternative alignments of the key helices 3 and 6 were “worse” with respect to the overlap of Na-ligands (cf. Additional file 1: Figures S2B and S3B). Hence, comparison of different superposition patterns indicates that the emergence of GPCR-like proteins from NRs may have been coupled with a specific “sliding” of the terminal 7^th^ helix by one turn towards the cytoplasmic side of the membrane relatively to the other six helices (see Additional file 1: Figure S10b). In either case, polar amino acid side chains of helix 7/G (boxed in Fig. 1f, Additional file 1: Figures S4, S10a, marked with arrows in S10b) form a “ladder” that allows passage of the Na^+^ ion across the membrane.

Aravind and co-workers [19] have suggested, based on a comparative genome analysis of the GPCR machinery, that the Last Eukaryotic Common Ancestor (LECA) could already contain both stand-alone 7TM receptors and 7TM receptors fused with the RGS-domains (from *r*egulators of *G*-protein *s*ignaling). According to their data, the RGS-fused versions, which could be present already in the LECA, resemble some 7TM bacterial receptors and also show a distant relationship with the metabotropic glutamate/receptor-like proteins (class C GPCRs) “suggesting that the later type could have emerged secondarily from a precursor of the former type” (quoted from [19]). This conclusion corroborates the suggestion that class C GPCRs could be traced directly to the eukaryotic root, separately of other GPCRs [10, 32, 33].

The available structures of Class C GPCRs [35, 36], although showing structural similarity to other GPCRs, contain no bound Na^+^ ion. Our structure-guided superposition of KR2, a class A GPCR δ-OR and a class C metabotropic glutamate receptor 1 [PDB: 4OR2] in Additional file 1: Figure S11a, while revealing the conservation of the Trp residue in helix 6, shows that the orientation of the Trp residue in KR2 is intermediate between its positions in these two GPCRs. While in class A GPCRs the Trp residue stabilizes the Na^+^ ion via a water bridge, in class C receptors, the Trp is turned away and additionally interacts with helix 5, which, as compared to other GPCR classes, sits deeper within the helical bundle [35, 36] and apparently stabilizes it. The structural and sequence alignments in Additional file 1: Figure S11, while supporting the common origin of all classes of GPCRs from MRs and revealing the conservation of the key Trp residue in the glutamate receptors, support the suggestion that the ancestors of class C GPCRs evolved independently from other GPCR classes [10, 19, 32, 33].

Hence, when taken together, the results of comparative genome analysis [18, 19], phylogenetic analyses [10, 32, 33] and our structural comparisons indicate that the LECA could contain 7TM receptors of at least two types. One type could be a stand-alone 7TM receptor that could spawn all classes of GPCRs except for class C; we suggest that this receptor developed from an ancient NR and had a Na^+^-binding site. Another 7TM receptor could contain a RGS domain, emerge from bacterial 7TM receptors (traceable, in turn, to bacterial rhodopsins [18]) and develop later into the class C GPCRs (glutamate receptors).

The alignments in Fig. 1f, Additional file 1: Figures S4, S10a offer a clue as to why previous attempts to find sequence similarity between MRs and GPCRs have failed: these attempts were focused on animal rhodopsins, which, as follows from these alignments, show the least resemblance to MRs. Non-opsin GPRCs, particularly those that have Na^+^-binding sites [25–28, 42], are much more similar to MRs (Fig. 1f, Additional file 1: Figures S4, S10a). The major sequence deviation of animal rhodopsins from non-opsin GPCRs might result from the need to re-accommodate the retinal moiety (see Fig. 2), which is bound to a unique Lys^7.43^ residue that is located on the same 7^th^ helix as retinal-binding Lys residues in MRs [2] but does not align with them either in the standard or alternative alignment (Fig. 1f, Additional file 1: Figures S4, S10a). Hence, our analysis, while indicating homology of MRs and GPCRs, supports the convergence in using retinal as a pigment in MRs and animal rhodopsins, see e.g. [86].

Until now, the search for similarities between functionally important residues of MRs and non-opsin GPCRs was hindered by the failure to find any function that would be common for these two groups of proteins. Their common ability to bind Na^+^ allowed us not only to produce structure-guided alignments, but also to look for further commonalities, such as the functionally important tilting of helix 6 in all these proteins.

In conclusion, the structure of the sodium-translocating microbial rhodopsin [40, 41] not only provides evidence on the common ancestry of two highly diverged (super)families of 7TM-containing proteins, it allows valuable insights into the evolution of structure and function of these molecular machines. Structure-guided alignments of GPCRs and MRs, obtained in this work, should be useful in establishing phylogenetic relations within separate families of these 7TM proteins.

## Reviewers’ comments

### Reviewer’s report 1: Prof. Oded Beja, Faculty of Biology, Technion - Israel Institute of Technology, Haifa, Israel

The authors are trying to solve the long-standing question of the evolutionary relation between G-protein coupled receptors and microbial rhodopsins. What exactly is the scenario suggested by the authors? (I am asking because there is no simple figure to explain).

SCENARIO? There were microbial rhodopsins (that use retinal, which one?). Some of them are sodium transporting rhodopsins. Then some of them lost the chromophore. Or did they lost transport activity first? Then the chromophore pocket is changing and another chromophore is entering (an opioid?). Then the pocket is changing again to become again a retinal pocket (which one?)……

Is this the most parsimonious explanation to what the authors observe? Can’t we simply say that the conservation of the sodium binding site is convergent evolution? As was used to explain the amazing structural similarities between the two families? (7TMs, Lysine at a similar position to bind retinal……)

The authors would do better if they supply a cartoon that explain their model (I mean their evolutionary model) and discuss why their suggestion is better than simply say it is just a convergent evolution of the sodium binding site.

Authors’ response: *We thank the reviewer for his comments; we took them into account while revising the manuscript and address them below.*

*An evolutionary scheme has been added as* Fig. 2. *In this scheme we emphasize by dashed arrows the ambiguity of evolutionary steps where losses/gains could take place either simultaneously or sequentially.*

*A convergence in recruiting retinal by MRs and visual rhodopsin, which is also supported by our structure guided alignments, follows, among others, from the observation that, upon the superposition of structures, the retinal-binding lysine residues do not overlap; they are located in different parts of helix 7. The situation with sodium ligands was different: they nicely overlapped when we superimposed the structures. We find it unlikely that in two unrelated 7TM proteins similarly placed residues in helices 3 and 7 would be involved in binding Na*^*+*^*just be chance.*

*In the revised manuscript we discuss in some more detail that the Na*^*+*^-*binding residues could be traced to the common ancestor of all GPCRs (except for class C GPCRs), which, supposedly, was present already in the Last Eukaryotic Common Ancestor (LECA). So the ability to bind Na*^*+*^*ion should have developed already at this early stage, where a recruitment of a Na*^*+*^-*binding MR for the receptor job is easy to imagine. For example, channelrhodopsin, which, being a MR, is found exclusively in eukaryotes, also contains the Na*^*+*^*ligands* (Fig. 1f, Additional file 1: Figures S4, S10).

*We have added to the main text the following paragraph: “Until now, the search for similarities between functionally important residues of MRs and GPCRs was hindered by the failure to find any function that would be common for these two groups of proteins. Their common ability to bind Na*^*+*^*allowed us not only to produce structure-guided alignments, but also to look for further commonalities, such as the functionally important tilting of helix 6 in all these proteins.”*

### Reviewer’s report 2: Prof. Gajendra P. S. Raghava, Bioinformatics Centre, CSIR-Institute of Microbial Technology, Chandigarh, India

In this manuscript entitled “Eukaryotic G protein-coupled receptors as descendants of prokaryotic sodium-translocating rhodopsins” written by “Daria N. Shalaeva, Michael Y. Galperin and Armen Y. Mulkidjanian, authors made an attempt to understand evolutionary relatedness between microbial rhodopsin (MR) and Eukaryotic GPCR (e-GPCR). In this study, authors compare newly solved structure of bacterial rhodopsin Krokinobacter eikastus (KR2) with structure of other MR and e-GPCR. Overall this is excellent contribution towards evolutionary relatedness between two superfamilies. I have following questions and/or suggestions.

Authors’ response: *We thank the reviewer for his comments; we address each of them below.*

1. In this study, authors used jFATCAT-rigid algorithm and PDBeFOLD server for structure comparison. In past number of methods have been developed for structure comparison, author should justify selection.

Authors’ response: *Despite the abundance of structural comparison methods available, very few servers matched our needs. We prefered the jFATCAT-rigid algorithm and the PDBeFOLD server because both these methods are relatively fast and allow similarity search against the entire PDB database. Additionally, in our experience, the SSM/PDBeFOLD server consistently produces good results when comparing distant proteins. The advantage of jFATCAT-rigid algorithm is the availability of pre-calculated superpositions for representative structures on the PDB web site. Finally, both this algorithms provided sequence alignments that reflected the results of structural superposition almost perfectly.*

*Along with the jFATCAT-rigid algorithm and the PDBeFOLD server, we have considered using other popular methods, particularly Dali server for a PDB-wide search. The list of matches when using KR2 structure [PDB: 4XTL] as template was similar to one produces by PDBeFOLD, and provided same pairwise alignments.*

*We also attempted to use MUSTANG program for pairwise comparison of KR2 with several e-GPCR structures. Unfortunately, this method failed to provide a productive superposition, producing RMSD values over 9 Å. In general, most tested algorithms generated reasonable alignments within MR and class A GPCR families but produced inconsistent inter-family alignments.*

2. In past number of methods have been developed for predicting GPCR and their class based on different types of composition like amino acid, dipeptide and PSSM composition. Authors should show overall and domain level compositional similarity between two superfamilies.

Authors’ response: *Following the suggestion of the Reviewer, we have attempted to analyze the differences in amino acid and dipeptide composition between GPCRs and MRs using the existing web-servers – GPCRsclass* [87]*, GPCR-Mpredictor* [88]*, PCA-GPCR* [89]*, and 7TMRmine* [90]*.*

*The server GPCRsclass *[87] (available at http://www.imtech.res.in/raghava/gpcrsclass/) *gave the same result “Your protein does not belong to Amine type of G-protein coupled Receptors” for every sequence we tried, both in the “Composition Based” and the “Dipeptide composition Based” modes. Surprisingly, even human Alpha-1A adrenergic receptor (UniProt: P35348) sequence, which was used as an example in the original paper* [87] *gave the same result. Thus we conclude that the server might be not functional at this time. The mirror site*http://bioinformatics.uams.edu/raghava/gpersclass/,* mentioned in the paper* [87], *was not accessible.*

*The online classifier for GPCRs GPCR-Mpredictor, described in* [88] (available athttp://111.68.99.218/gpcr-mpredictor/),* could not be reached either.*

*We also considered the server “PCA-GPCR: Prediction of G-protin-coupled receptor classes” (available at*http://www1.spms.ntu.edu.sg/~chenxin/PCA_GPCR/) *which takes into account amino acid composition and dipeptide composition along with many other sequence features* [89]. *According to the sequence-based prediction of this server, the sodium-translocating rhodopsin from Krokinobacter eikastus* (KR2) is a GPCR protein and belongs to the “Family: Vomeronasal receptors (V1R & V3R), SubFamily: Vomeronasal receptors V1RJ & VIRK”. This method assigned several other MRs to various GPCR families, but gave no numerical scores or estimates of prediction reliability. Our additional test has, however, shown that this server assigns GPCR family classification even to cytochrome *c, a small, globular, water soluble protein with dozens of charged residues on its surface, which is definitely unrelated to GPCRs.*

*Finally, the 7TMRmine server* [90], *available at*http://bioinfolab.unl.edu/emlab/7tmr, *specifically excluded archaeal/bacterial/fungal opsins from sampling and treated them as false positives. The analysis of the KR2 sequence at this web server resulted in its recognition as potential GPCR by some of the employed methods but not the others.*

*In summary, while the very idea of using amino acid, dipeptide and PSSM composition for determining evolutionary relations between different proteins seems to be promising, none of the available servers could help us in establishing the relations between Na*^*+*^*-translocating rhodopsin and GPCRs.*

3. Authors should show multiple sequence alignment using alignment viewers like JalView.

Authors’ response: *We believe that specificity of our task justifies introduction of custom coloring scheme to highlight patters of Na*^*+*^*binding residues and other polar residues which can support Na*^*+*^*ion passage between the transmembrane helices of the proteins. Still, on the request of Reviewer, we provide, in the revised version of the manuscript, a representation of the alignment that was created by Jalview* [91] *with the use of the Taylor coloring scheme* [92] *(new Additional file 1: Figures S4b, S8 and S9 ).*

### Reviewer’s report 3: Dr L. Aravind, NCBI, NLM, National Institutes of Health

Shalaeva et al. compare the structures of microbial rhodopsins (MRs) and G-protein coupled receptors (7TM hereafter) to propose a common functional mechanism centered on a conserved aromatic residue in TM helix 6. There has been a long-standing discussion regarding the common origin of the 7TM receptors of eukaryotes and bacterial 7TM proteins. While there have been former proposals for a convergent origin, this reviewer holds that the weight of the evidence favors a common origin for these proteins, perhaps with a larger radiation of bacterial 7TM receptors. The structural comparisons and the evidence presented by the authors make a strong case of the common origin of the MRs and eukaryotic 7TM receptors. The functional aspects uncovered as a result of this comparison certainly merit future attention in wet-lab studies.

Authors’ response: *We thank the Reviewer for his insightful comments that helped us a lot upon revising the manuscript.We are very happy that the Reviewer considers our case of the common origin of the MRs and eukaryotic 7TM receptors to be strong.*

### However, the authors should consider certain issues in their functional discussion:

1) It was earlier demonstrated that eukaryotic 7TM receptors working with heterotrimeric G-proteins come in two basic types ([19]; the authors may want pay closer consideration to this paper as it is relevant to their discussion in more than one way). First, those that are fused to the intracellular RGS domains and function as GTPase-activating proteins (GAPs) for their GTPase #-subunit partners. Second those that act as GDP-GTP exchange factors (GEFs) for their G# subunits. These seem to coevolve with the selection imposed by the GTP hydrolysis rate constants of the G#s. The functional analysis by the authors focuses on the second type and seems to ignore the first type. Given that these types were present from very early in eukaryotic evolution it is important to examine if the conserved features recovered by the authors are relevant only to those that function as GEFs or also extend to those that work as GAPs. Either way it affects the final evolutionary reconstruction.

2) Several eukaryotic lineages have lost heterotrimeric G proteins but retain 7TM receptors clearly related to the GPCRs (see discussion on this in [19]). Moreover, even in lineages with G#s there is good evidence for G-protein-independent signaling via 7TM receptors. Hence, it is possible that there was always a parallel G#-independent signaling track. The authors need discuss this better because MRs work independently of G# in prokaryotes. Thus conserved features shared by them could represent a hold-over of the more ancient G#-independent signaling which still exists in eukaryotes but is merely reused in the presence of G#s

Authors’ response: *We fully agree with these comments of the Reviewer. Still, even in the revised manuscript, we do not discuss the signal chain components beyond the 7TM receptors proper. The main reason is that these components do not have obvious counterparts among proteins that interact with MRs, and the topic of our paper is limited to the comparison of the 7TM-MRs with 7TM-GPCRs and GPCR-like proteins.*

*However, we have considered the articles by Anantharaman and co-workers* [18, 19] *very carefully and fully agree with the Reviewer that they are relevant to our work in more than one way. This relevance is now discussed in a separate new section that sorts out the relation between diverse bacterial 7TM proteins and different classes of GPCRs based on their ability/inability to bind Na*^*+*^.

## Additional file

Additional file 1:
**Methods, supplementary table and figures.** (PDF 1775 kb)

## Abbreviations

cAMP: 3′–5'-cyclic adenosine monophosphate
δ-OR: Na^+^-bound δ-opioid receptor
GPCR: G-protein coupled receptor
KR2: Na^+^-transporting rhodopsin from the bacterium *Krokinobacter eikastus*
LECA: Last Eukaryotic Common Ancestor
LUCA: Last Universal Cellular Ancestor
MR: Microbial rhodopsin
NR: Na^+^-translocating microbial rhodopsin
RGS: Regulators of G-protein signaling
RMSD: Root-mean-square deviation (of the protein backbone atoms)
TM: Transmembrane
