# Supplementary material for: Eukaryotic G protein-coupled receptors as descendants of prokaryotic sodium-translocating rhodopsins
Source: Biol Direct. 2015 Oct 15;10:63. doi: 10.1186/s13062-015-0091-4 (PMC4608122; doi:10.1186/s13062-015-0091-4)
Supplement: Additional file 1: — Methods, supplementary table and figures. (PDF 1775 kb) [file 13062_2015_91_MOESM1_ESM.pdf]

Additional File 1.

Supplementary Information

to the article "Eukaryotic G protein-coupled receptors as descendants of prokaryotic sodium-translocating rhodopsins"

by Daria N. Shalaeva, Michael Y. Galperin, and Armen Y. Mulkidjanian

To investigate the structural similarity between Na^+-translocating bacterial rhodopsin (hereafter KR2, PDB: 4XTL) [1] and other 7TM proteins, as described in the main text, we aligned KR2 with the δ-type opioid receptor (hereafter δ-OR, PDB: 4N6H [2]) using the secondary structure matching (SSM) method implemented on the PDBeFold server [3] (http://www.ebi.ac.uk/msd-srv/ssm/). The corresponding sequence alignment is presented on Figure S1.

In addition, we used the "3D-similarity" option on the web server of the Protein Data Bank (PDB) [4], which provides protein structure alignments, pre-calculated by the jFATCAT-rigid algorithm [5, 6]. In the list of structures similar to KR2, microbial rhodopsins (MRs) were followed by structures of diverse G-protein coupled receptors (GPCRs) including several Na^+-bound GPCRs, namely the proteinase-activated receptor 1 (PDB: 3VW7 [7], RMSD 4.27 Å, P-value 1.53E-8), A_{2A} adenosine receptor (PDB: 4EIY [8], RMSD 4.16 Å, P-value 1.84E-8), β_1 adrenergic receptor (PDB: 4BVN [9], RMSD 4.6 Å, P-value 2.37E-8), and the δ-OR (PDB: 4N6H [2], RMSD 4.49 Å, P-value 2.38E-8). Despite the overall similarity between the structures of these GPCRs (see Table S1 for RMSD values) and particularly their Na^+ binding sites [10], the GPCRs showed two distinct alignment patterns with the structure of KR2 (cf. Fig. S2 and S3). One pattern, exemplified by the alignment of KR2 with the structure of δ-OR[2], provided a match for all seven helices (Figure S2a) and superposition of the conserved tryptophan residues in helix 6 (Figure S2b) and was very similar to the alignment that we obtained by using the SSM/ PDBeFold method [3], see Fig. 1. Another pattern is exemplified by the alignment of the KR2 structure with the A_{2A} adenosine receptor (PDB: 4EIA) [8], see Fig. S3. Here, only six helices out of seven could be aligned (Fig. S3a) and the conserved Trp residues were shifted by one helix turn in the sequence alignment (Fig. S3b). With different GPCRs, the jFATCAT-rigid algorithm [5, 6] produces either one or the other alignment pattern, see the PDB web site http://www.rcsb.org/pdb/explore/structureCluster.do?structureId=4XTL.

Overall, we considered the alignment pattern that is shown in Fig. 1, S1, and S2 to be the more reliable one because a) it covered the entire protein and b) it was better compatible with the almost universal conservation and functional relevance of the Trp residue in helix 6 (see the main text). Accordingly, we used this alignment in the subsequent work. Still, both structural alignment patterns should be taken into account in further studies of the evolutionary relations between GPCRs and MRs.

Visualization of structures was performed with PyMOL [11].

Table S1. Structures of MRs and GPCRs used for the superposition. The structures with names shown in bold were used for the initial pairwise superposition, which was then expanded to include a wide range of GPCR and MR structures, by aligning them with 4XTL or 4N6H, respectively. Alignment properties are given as provided by the jFATCAT algorithm on the PDB web site [5, 6]. All GPCRs listed belong to class A, with exception of Class C glutamate receptor.

PDB	RMSD, Å	Number of aligned residues	Protein name	Ref.
Microbial rhodopsins, aligned to 4XTL				
**4XTL**	**N/A**	**N/A**	**Sodium pumping rhodopsin (KR2)**	[1]
3QBG	3.30	231	Halorhodopsin	[12]
3QAP	2.97	216	Sensory rhodopsin II	[13]
3UG9	3.00	221	Channelrhodopsin (ChR1 and ChR2 chimera) **	[14]
2JAF	3.20	223	Halorhodopsin	[15]
5AX0	3.22	225	Rhodopsin I	unpublished
4JQ6	2.02	196	Blue-light absorbing proteorhodopsin	[16]
4HYJ	2.39	233	Proton-pumping bacteriorhodopsin	[17]
3DDL	2.43	247	Xanthorhodopsin	[18]
G-protein coupled receptors, aligned to 4N6H				
**4N6H**	**N/A**	**N/A**	**δ-opioid receptor (δ-OR)**	**[2]**
4DKL	0.67	216	μ-opioid receptor	[19]
4BVN	1.96	169	β1-adrenoceptor	[9]
2RH1	1.55	184	β2-adrenoreceptor	[20]
3PBL	3.12	218	Dopamine D3 receptor	[21]
3VW7	2.39	197	Protease-activated receptor 1	[22]
4EIY	2.99	204	A(2A) adenosine receptor	[8]
4BUO	2.67	223	Neurotensin receptor 1	[23]
4IAR	1.69	191	Serotonin receptor	[24]
3V2Y	3.54	198	Lipid G protein-coupled receptor	[25]
4MQS	1.98	181	M2 muscarinic acetylcholine receptor	[26]
4MBS	1.75	226	CCR5 chemokine receptor	[27]
1U19	2.85	193	Visual pigment rhodopsin	[28]
4OR2	3.01*	202*	Glutamate receptor 1 (Class C GPCR)	[29]

*The structural superposition of the class C and class A GPCR structures was constructed using the PDBeFold server [30], see Figure S11 for details.

** The structure of chimera protein was used as a template to include in the alignment (Figure 1D, main text) the sequence of the channelrhodopsin 2 (ChR2) from *Chlamydomonas reinhardtii* (UniProt Q8RUT8_CHLRE).

```
     23               40                        60                  80
     |      |    .    |    .    |        .      |    .    |    .    |    .
4XTL IAYQFTSHILTLGYAVMLAGLLYFILTIKNV----DKKF--QMSNILSAVVMVSAFLLLYAQAQNWTSSF
     ........|..|..||...|||..:|....:    ..|.  .......|.....|...|..|...:....
4N6H SSLALAIAITALYSAVCAVGLLGNVLVMFGIVRYTKMKTATNIYIFNLALADALATSTLPFQSAKYLMET
     |     |    .    |    . |       .    |    .    |    |    |    .    |
     44              60     1.50         80             2.50 100

                  100                 120                             140
        |    .    |    .    |    .    |      .    |                 .    |
4XTL TFNEEVGRYFLDPSGDLFNNGYRYLNWLIDVPMLLFQI--LFVVSLT---------TS----KFSSVRNQ
                 |.|::.................:|.:  :  ....         ..    |...:...
4N6H W-----------PFGELLCKAVLSIDY-YNMFTSIFTLTMMSVDRYIAVCHPVKALDFRTPAKAKLINIC
                  .    |    .     |   |    |    .|   |    .    |    .    |
                       120        3.35     140   3.50          160
                                      3.39

                                    160                  180
        .    |    .                 |    .    |    .     |    .    |    .
4XTL FWFSGAMMIITGYIG-------------QFYEVSNLTAFLVWGAISSAFF-FHILWVMKKVINEGKEG--
     .|.......:...:.             ..:  .......|.......|. ...:.:......... .
4N6H IWVLASGVGVPIMVMAVTRPRDGAVVCMLQFPSPSWYWDTVTKICVFLFAFVVPILIITVCYGLMLLRLR
      | .    |    .    |    .    |    .    |    .    |    |    |    .    |
      4.50   180                 200                 220  5.50           240

                      200                 220                 240
                      |    .    |    .    |    .    |    .    |    .    |
4XTL -------------ISPAGQKILSNIWILFLISWTLYPGAYLMPYLTGVDGFLYSEDGVMARQLVYTIADV
                  ......:....:...|.:.|.......:...|..  ........|.|..|...:...
4N6H SVRLLSGSKEKDRSLRRITRMVLVVVGAFVVCWAPIHIFVIVWTLVD--IDRRDPLVVAALHLCIALGYA
        .    |    .    |    .    |   |.|   |    .      |    .    |    .
                       260        6.48 6.50                      300

            260
       .    |    .    |
4XTL SSKVIYGVLLGNLAITLS
     . ..:..||...|.....
4N6H -NSSLNPVLYAFLDENFK
      |   ||  |      .
     7.45 7.507.53
         7.49
```

Figure S1. Sequence alignment of KR2 (PDB: 4XTL) and δ-OR (PDB: 4N6H), manually derived from the structural superpositions in Fig. 1, panels A–E.

The structural alignment in Fig. 1 was used to manually curate the sequence alignment provided by the jFATCAT software [5, 6] on the PDB server (Fig. S2b) by checking the positions and orientations of the Na$^+$ -binding residues, which resulted in the introduction of a few structurally justified gaps (Fig. S1). The Na$^+$-binding residues are shown in red, the reference 50th residues for the Ballesteros-Weinstein numbering scheme [31, 32] are shown in bold font. In helices 1 and 2, the highly conserved Na$^+$ ligands Asn$^{\mathbf{1.50}}$ and Asp$^{\mathbf{2.50}}$ of GPCRs do not have functional counterparts in either KR2 or other MRs; the helices 4 and 5 do not carry Na$^+$ ligands. Thus, we primarily considered helices 3, 6 and 7 for the multiple alignment construction (Fig. S4).

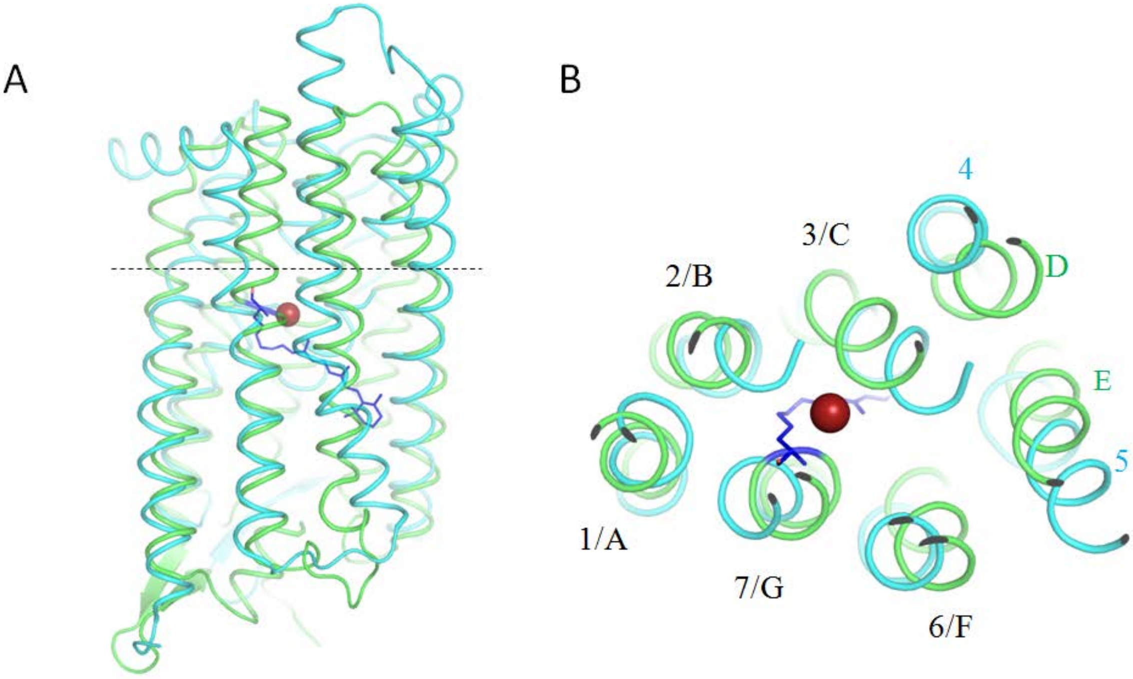

Figure S2a. Structural superposition of KR2 (PDB: 4XTL) with δ-OR (PDB: 4N6H).

The pre-calculated structural superposition with P-value 2.38 E-8, based on the jFATCAT-rigid algorithm [5, 6], is available from the PDB web site (http://www.rcsb.org/pdb/explore/structureCluster.do?structureId=4XTL).
The KR2 structure is in green, the δ-OR structure is in blue. The Na^+ ion in δ-OR is shown as a red sphere. The retinal molecule bound to the KR2 lysine residue is shown in dark blue. A, side view; B, top view from the extracellular side, the structures were cut along the dashed line.

```
     23               40                        60                  80
     |      |    .    |    .    |        .      |    .    |    .    |    .
4XTL IAYQFTSHILTLGYAVMLAGLLYFILTIKNV----DKKF--QMSNILSAVVMVSAFLLLYAQAQNWTSSF
     ........|..|..||...|||..:|....:    ..|.  .......|.....|...|..|...:....
4N6H SSLALAIAITALYSAVCAVGLLGNVLVMFGIVRYTKMKTATNIYIFNLALADALATSTLPFQSAKYLMET
     |     |    .    |    . |       .    |    .    |    |    |    .    |
     44              60     1.50         80             2.50 100

                  100                 120                             140
        |    .    |    .    |    .    |      .    |                 .    |
4XTL TFNEEVGRYFLDPSGDLFNNGYRYLNWLIDVPMLLFQI--LFVVSLT---------TS----KFSSVRNQ
                  ...  ................:|.:  :  ....         ..    |...:...
4N6H W------------PFGELLCKAVLSIDYYNMFTSIFTLTMMSVDRYIAVCHPVKALDFRTPAKAKLINIC
                  .    |    .     |   |    |    .|   |    .    |    .    |
                       120        3.35     140   3.50          160
                                      3.39

                                    160                  180
        .    |    .                 |    .    |    .     |    .    |    .
4XTL FWFSGAMMIITGYIG-------------QFYEVSNLTAFLVWGAISSAFF-FHILWVMKKVINEGKEG--
     .|.......:...:.             ..:  .......|.......|. ...:.:......... .
4N6H IWVLASGVGVPIMVMAVTRPRDGAVVCMLQFPSPSWYWDTVTKICVFLFAFVVPILIITVCYGLMLLRLR
      | .    |    .    |    .    |    .    |    .    |    |    |    .    |
      4.50   180                 200                 220  5.50           240

                      200                 220                 240
                      |    .    |    .    |    .    |    .    |    .    |
4XTL -------------ISPAGQKILSNIWILFLISWTLYPGAYLMPYLTGVDGFLYSEDGVMARQLVYTIADV
                  ......:....:...|.:.|.......:...|..  ........|.|..|...:...
4N6H SVRLLSGSKEKDRSLRRITRMVLVVVGAFVVCWAPIHIFVIVWTLVD--IDRRDPLVVAALHLCIALGYA
        .    |    .    |    .    |   |.|   |    .      |    .    |    .
                       260        6.48 6.50280                   300

            260
       .    |    .    |
4XTL SSKVIYGVLLGNLAITLS
     . ..:..||...|.....
4N6H N-SSLNPVLYAFLDENFK
     |    ||  |      .
     7.45 7.507.53
          7.49
```

Figure S2b. Sequence alignment based on the structural superposition of KR2 (PDB: 4XTL) and δ-OR (PDB: 4N6H) by the jFATCAT-rigid algorithm [5, 6].
Pre-calculated sequence alignment is available from the PDB web site: (http://www.rcsb.org/pdb/explore/structureCluster.do?structureId=4XTL).
The number of aligned residues: 225, identity: 9.40%, similarity: 20.81%. The Na^+-binding residues are shown in red, the reference 50th residues for the Ballesteros-Weinstein numbering scheme [31, 32] are shown in bold font.

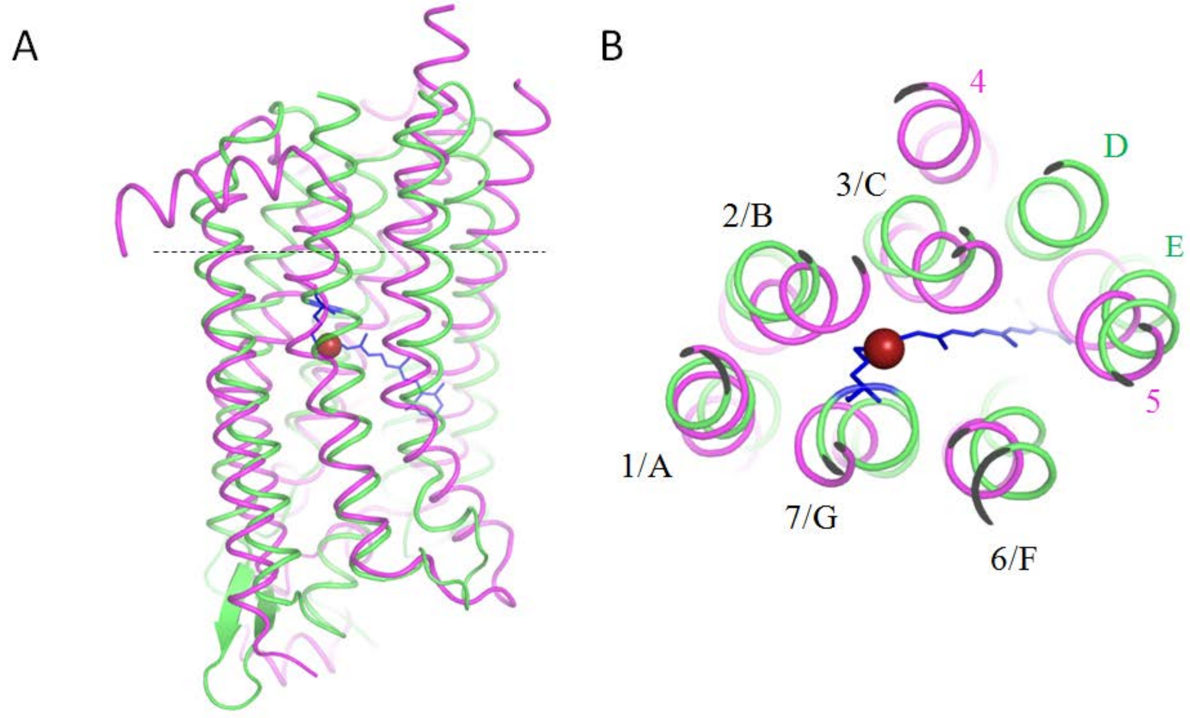

Figure S3a. Structural superposition of KR2 (PDB: 4XTL) and A_{2A} adenosine receptor (PDB: 4EIY).

Pre-calculated alignment with P-value of 1.84 E-8, based on the jFATCAT-rigid algorithm [5, 6], is available from the PDB web site (http://www.rcsb.org/pdb/explore/structureCluster.do?structureId=4XTL).

The KR2 structure is in green, the structure of the A_{2A} adenosine receptor is in magenta. The Na^+ ion in the A_{2A} adenosine receptor is shown as a red sphere. The retinal molecule bound to KR2 lysine residue is shown in dark blue. A, side view; B, top view from the extracellular side, the structures were cut along the dashed line.

```
      21                 40                  60                  80
      |        |    .    |    .    |    .    |    .    |    .    |    .    |
4XTL  SEIAYQFTSHILTLGYAVMLAGLLYFILTIKNVDKKFQMSNILSAVVMVSAFLLLYAQAQNWTSSFTFNE
      ..........:........:.|.......:..........|.........................|
4EIY  PIMGSSVYITVELAIAVLAILGNVLVCWAVWLNSNLQNVTNYFVVSLAAADIAVGVLAIPFAITIST---
      |       |    .    |   |.    |    .    |    .    | |  .    |    .
      2                 20  1.50            40          2.50    60

               100                      120                             140
          .    |       .    |    .    |    .    |    .              |    .
4XTL  EVGRYFLDPSG---DLFNNGYRYLNWLIDVPMLLFQILFVVSLTTSKFSSV----------RNQFWFSGA
            .....   .||...:........:..||...:........               .   .   .
4EIY  ------GFCAACHGCLFIACFVLVLTQSSIFSLLAIAIDRYIAIRIPLRYNGLVTGTRAKGIIAICWVLS
             |    .    |    .    ||   .    | |  .    |    .    |    .   ||
                       80         3.39       3.50              120      4.50

                                           160                     180
        |    .                             |    .    |      .    |    .    |
4XTL  MMIITGYIGQFY-------------------------EVSNLTAFLVWGAIS--SAFFFHILWVMKKVIN
        |.  .   ..                         :|..:..........  .......|.|..::..
4EIY  FAIGLTPMLGWNNCGQPKEGKNHSQGCGEGQVACLFEDVVPMNYMVYFNFFACVLVPLLLMLGVYLRIFL
        .    |    .    |    .    |    .    |    .    |    .   ||    .    |
             140                 160                 180      5.50

                      200                   220                     240
       .    |    .    |    .    |    .      |    .    |    .    |    .
4XTL  EGK---EGISPAGQKILSNIWILFLISWTLYPGAYLMPYLT--GVDGFLYSEDGVMARQLVYTIADVSSK
      ..:   .............:.|:..:....:...::....|  ..|....................|. .
4EIY  AARRQLRSTLQKEVHAAKSLAIIVGLFALCWLPLHIINCFTFFCPDCSHAPLWLMYLAIVLSHTNSVV-N
        .      .    |    .    |    .| | |    .    |    .    |    .    |    |
                    230          6.48 6.50                  270    7.45  7.49

          260
          |    .
4XTL  VIYGVLLGNLAI
      ............
4EIY  PFIYAYRIREFR
      |  | |    .
    7.50 7.53
```

Figure S3b. Sequence alignment based on the structural superposition of KR2 (PDB: 4XTL) and A_{2A} adenosine receptor (PDB: 4EIY) by the jFATCAT-rigid algorithm [5, 6]. Pre-calculated sequence alignment is available from the PDB web site (http://www.rcsb.org/pdb/explore/structureCluster.do?structureId=4XTL). The number of aligned residues: 219, identity: 5.14% , similarity: 19.18%. The Na^{+}-binding residues are shown in red, the reference 50th residues for the Ballesteros-Weinstein numbering scheme [31, 32] are shown in bold font.

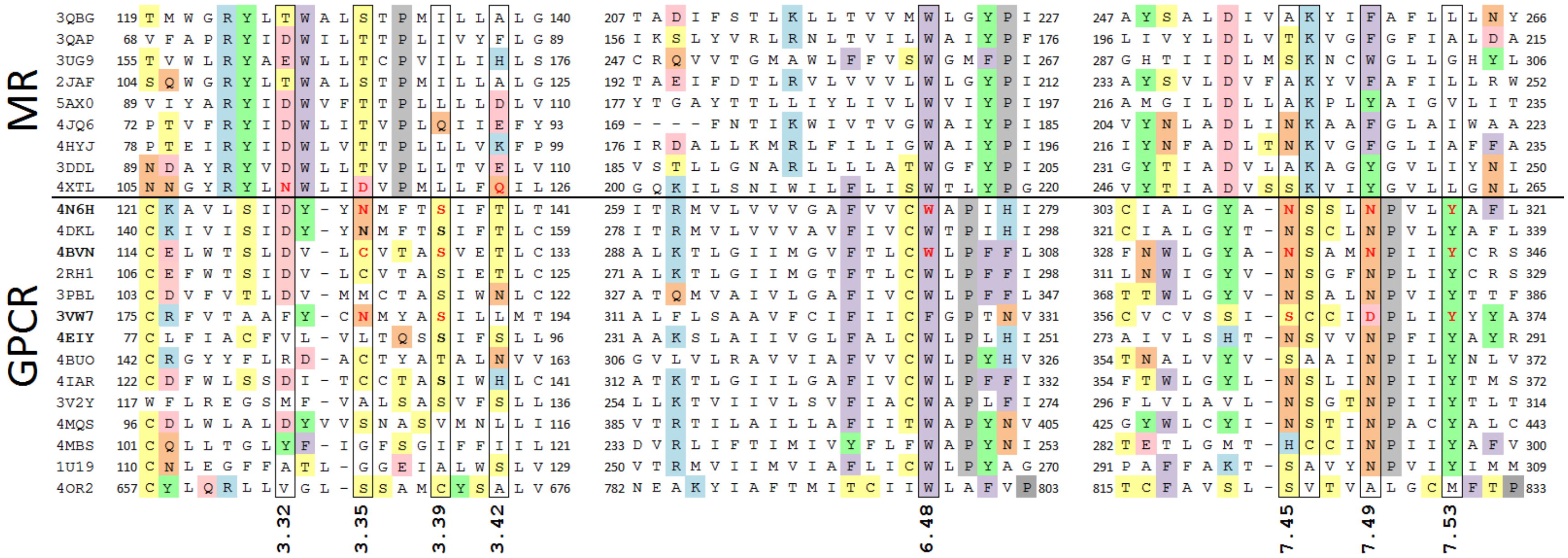

Figure S4a. Multiple sequence alignment of Na^{+}-binding regions of MRs and GPCRs (see Table S1 for the list of all structures). The alignment is based on the superposition of KR2 and δ-OR structures (Fig. 1, S1) All MR structures were aligned to the KR2 structure (PDB: 4XTL [1]), and all GPCR structures were aligned to δ-OR (PDB: 4N6H [2]) using the results produced by the jFATCAT-rigid algorithm. The resulting sequence alignments were inspected manually and corrected in a few cases to ensure the best matching to the respective structural superpositions (cf. Fig, A2b with Fig. S3b, A4). Only helices 3, 6 and 7 are shown. The boxes indicate the positions corresponding to known Na^{+}-binding residues in GPCRs. The bold red font indicates those amino acid residues that are involved either in the binding of Na^{+} in GPCRs [2, 8-10] or in coordinating the imino group of the Schiff base and, supposedly, in accommodating the Na^{+} ion in the KR2 structure [1, 33, 34]. As noted in the main text, the deprotonated Schiff base of the Lys255 residue in KR2 can serve as a Na^{+} ligand, which would justify matching this residue with $Ser^{7.46}$ of GPCRs. Aromatic amino acids are shaded violet, proline is shaded gray, tyrosine is shaded green, other residues capable of forming hydrogen bonds are shaded by different colors depending on their charge. Being aware of the subtle structural deviations (bulges) between Class A and Class C GPCRs described earlier [32], we still provide a non-gapped sequence alignment because our structural alignment was not exact enough to justify the respective gaps.

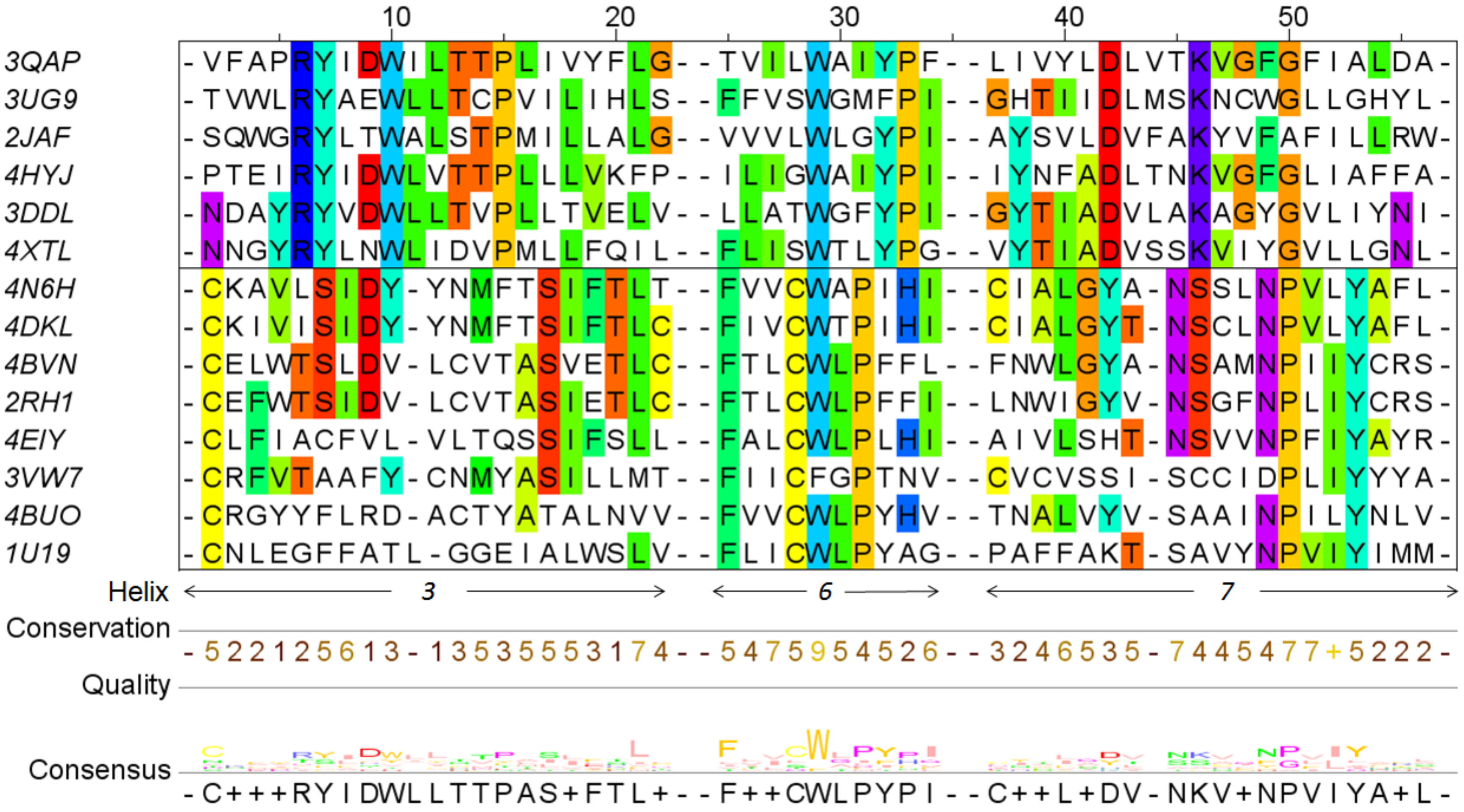

Figure S4b. Multiple sequence alignment of Na^{+}-binding regions of MRs and GPCRs (see Table S1 for the list of all structures). Alignment visualization and annotation (Conservation, quality and consensus fields) were created using Jalview software [35]. The Taylor coloring scheme [36] was applied separately for two groups: MRs and GPCRs with 20% identity threshold.

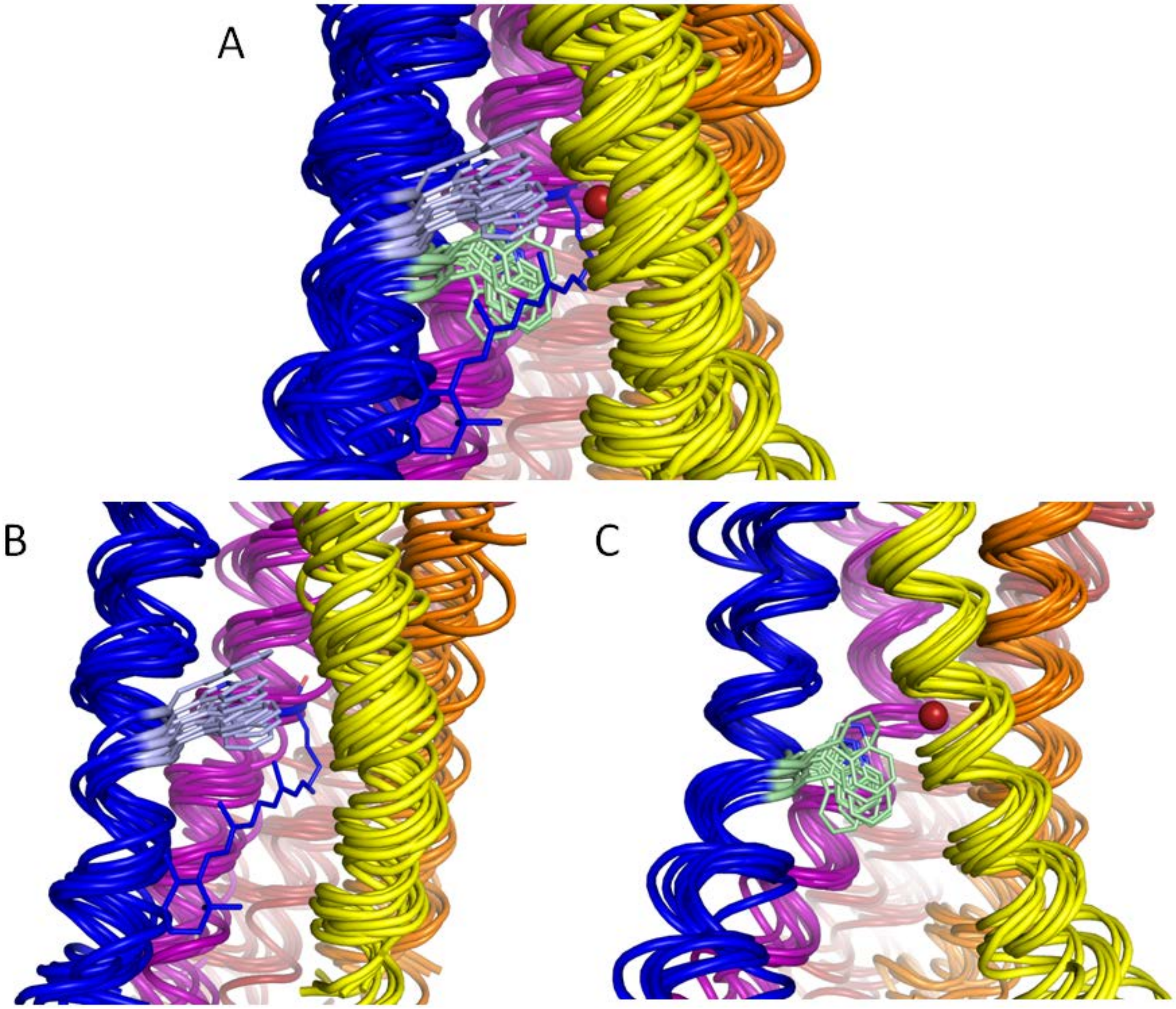

Figure S5. Varying orientation of the conserved Trp$^{6.48}$ residue of helix 6 (helix F of MRs). A, Multiple structural superposition of GPCRs and MRs (see Table S1 and Fig. 1) that shows variable orientation of the Trp residues in different structures; note the matching positions of the Cα atoms of Trp$^{\mathbf{6.48}}$ in MRs and GPCRs. Helices 1/A (red), 2/B (orange), 3/C (yellow), 6/F (blue), and 7/G (purple) are shown in cartoon representation, the Trp residues are shown as sticks, light-blue in MRs and light-green in GPCRs. The Na^+ ion in the δ-OR structure is shown as a red sphere. The retinal molecule bound to KR2 lysine residue is shown in dark blue.
B, multiple superposition of MRs aligned to KR2 (PDB: 4XTL). C, multiple superposition of GPCRs aligned to δ-OR (PDB: 4N6H)

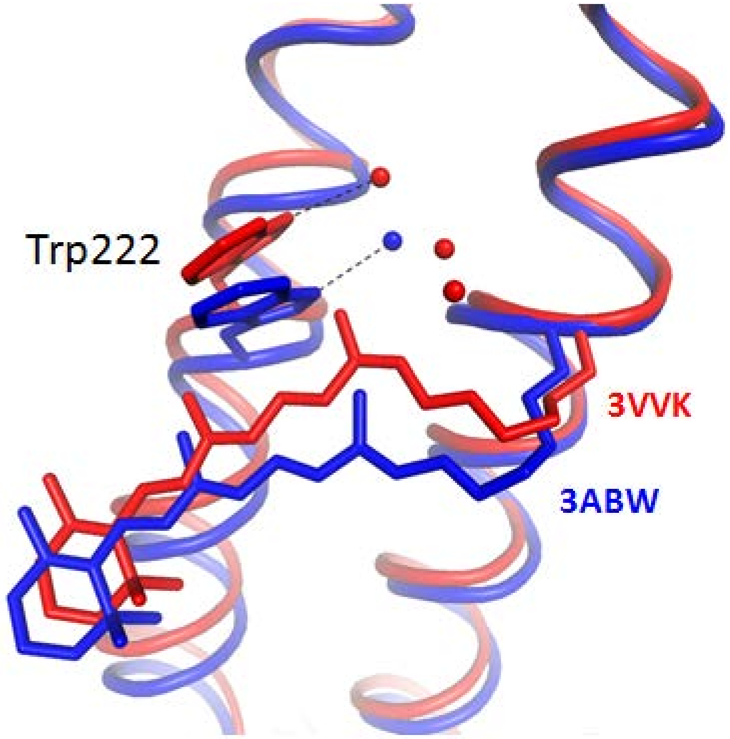

Figure S6. **Conformation of the conserved Trp residue of helix F of MRs.** Coupling between the conformation of the retinal, the orientation of the conserved Trp222 and the tilt of helix F in MRs, as shown for the azide-bound halorhodopsin from *Natronomonas pharaonis* [37]. The unphotolyzed state (PDB: 3ABW) is colored blue, while the reactive state (PDB: 3VVK) is shown in red. The water molecules are shown as small spheres.

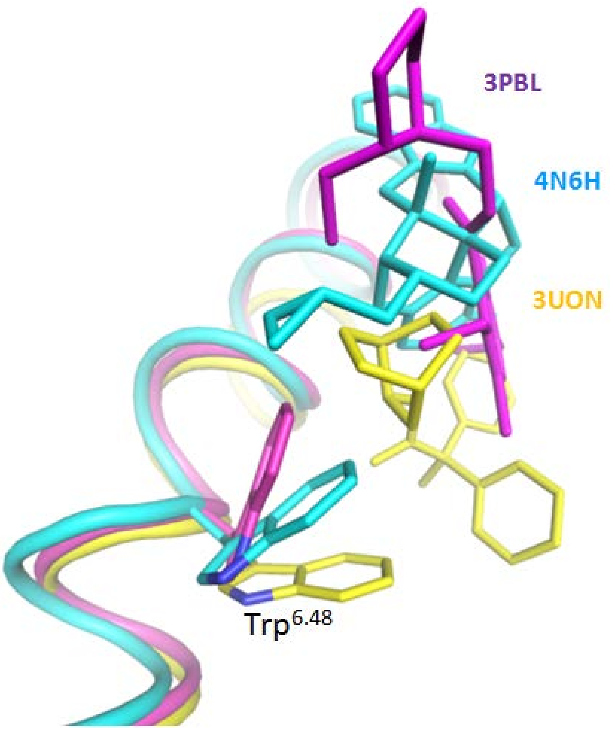

Figure S7. Conformations of Trp$^{6.48}$ in GPCRs bound with different signaling molecules. The orientation of the side chain of Trp$^{6.48}$ depends on the size and position of the signaling molecule as follows from the structural superposition of the dopamine D3 receptor [21] (PDB: 3PBL [21][21], magenta), the δ-opioid 7TM receptor [2] (PDB: 4N6H, cyan), and the M2 muscarinic receptor [38] (PDB: 3UON, yellow)

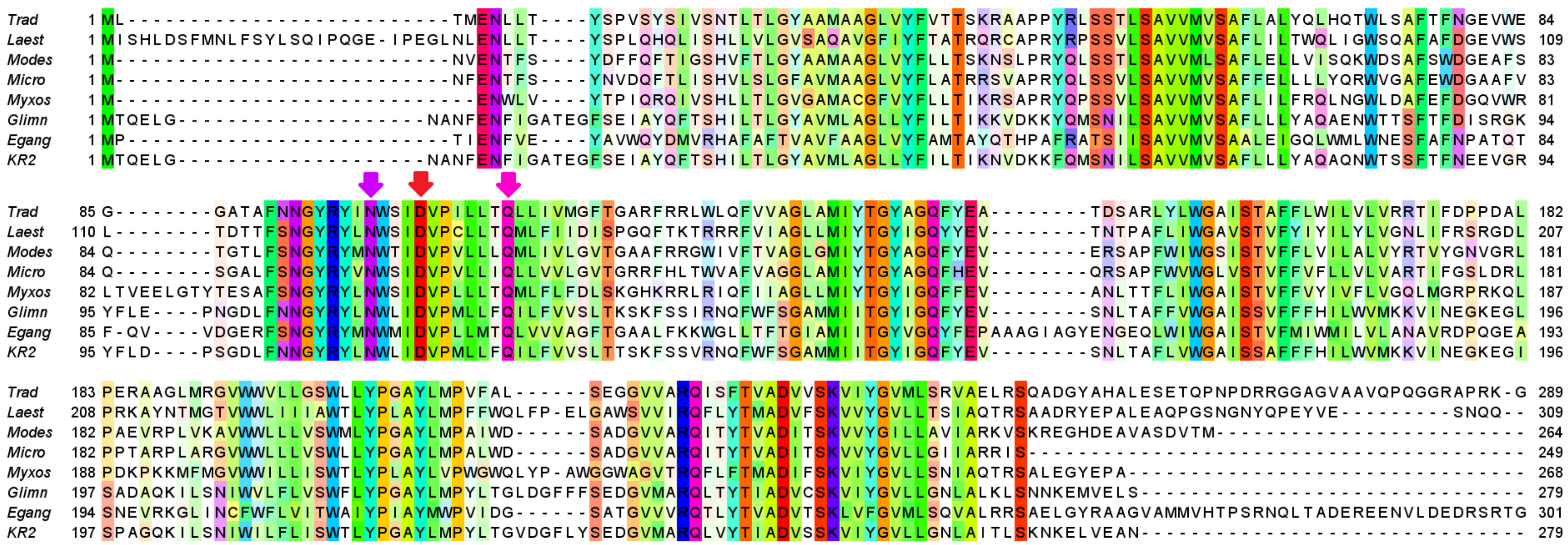

Figure S8. Multiple sequence alignment of rhodopsin-like proteins containing the Na^+ binding motif NDQ from various Bacteria. The residues of the “NDQ” triad, which is characteristic for sodium-pumping rhodopsins [39], are marked by arrows. The Taylor coloring scheme [36] was applied with the 30% identity threshold. The sequences are from the following organisms:

Trad – (GenBank: ADI15362.1) rhodopsin from *Truepera radiovictrix* (*Deinococcus-Thermus; Deinococci*)

Laest – (GenBank: ERT09063.1) putative membrane protein from *Lyngbya aestuarii* (*Cyanobacteria; Oscillatoriophycideae*),

Modes – (RefSeq: WP_036336161.1) partial sequence of rhodopsin from *Modestobacter sp*. (*Actinobacteria; Actinobacteria*),

Micro – (RefSeq: WP_036345212.1) partial sequence of rhodopsin from *Micromonospora* sp. (*Actinobacteria; Actinobacteria*),

Myxos – (RefSeq: WP_036488954.1) hypothetical protein from *Myxosarcina sp.* GI1 (*Cyanobacteria; Pleurocapsales*),

Glimm – (RefSeq: WP_040506994.1) rhodopsin from *Gillisia limnaea* (*Bacteroidetes; Flavobacteriia*),

Egang – (GenBank: KLE31050.1) rhodopsin from *Erythrobacter gangjinensis* (*Proteobacteria; Alphaproteobacteria*),

KR2 – (UniProt N0DKS8_9FLAO) sodium pumping rhodopsin from *Dokdonia eikasta* (*Bacteroidetes; Flavobacteriia*).

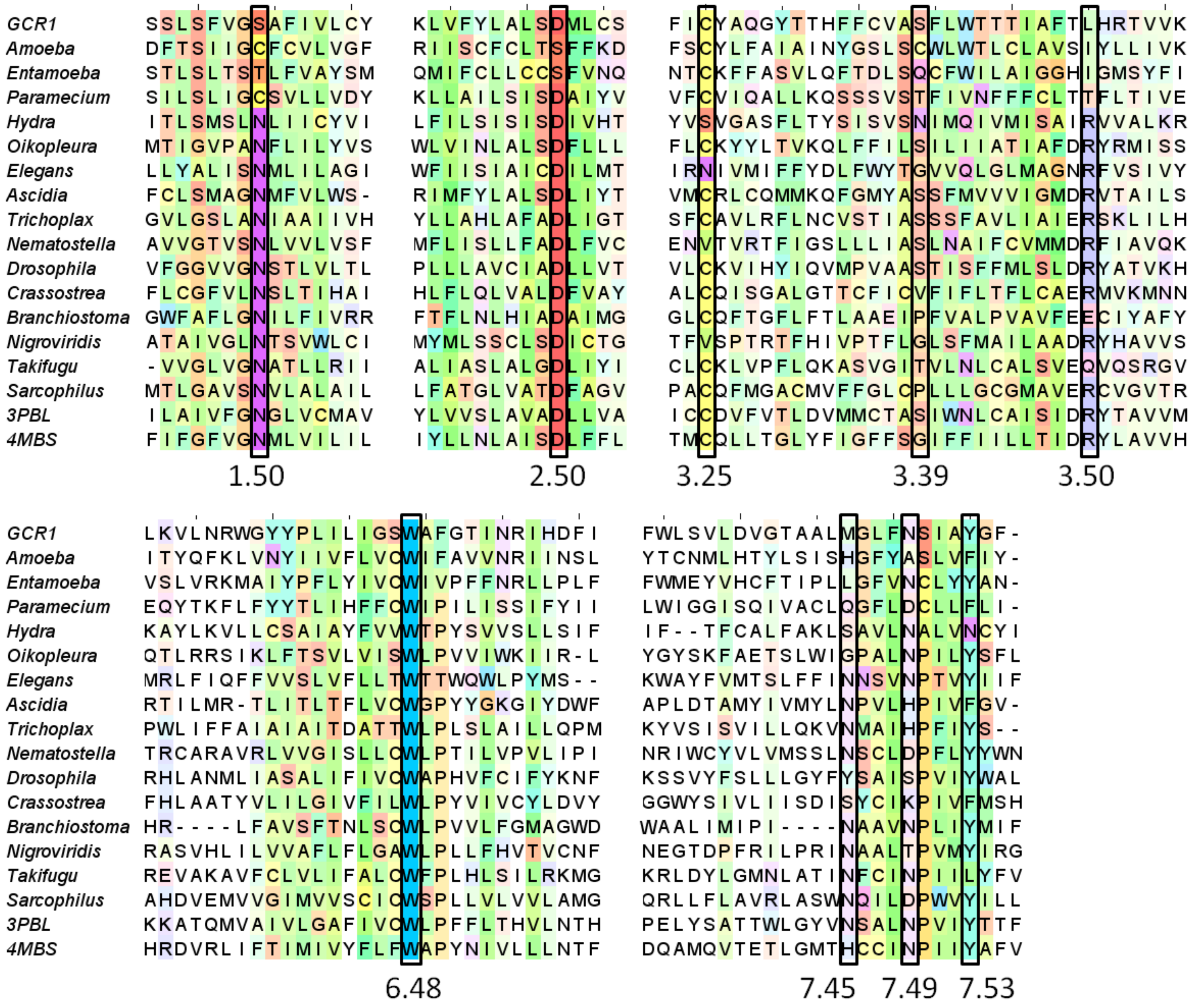
GCR1 SSLSFVGSSAFIVLCY KLVFYLALSDMLCS FICYAQGYTTHFFCVASFLWTTTIAFTLHRTVVK
Amoeba DFTSIIGCFCVLVGF RIISCFCLTSFFKD FSCYLFAIAINYGSLSCWLWTLCLAVSIYLLIVK
Entamoeba STLSLTSTLFVAYSM QMIFCLLCCSFVNQ NTCKFFASVLQFTDLSQCFWILAIGGHIGMSYFI
Paramecium SILSLIGCSVLLVDY KLLAILSISDAIYV VFCVIQALLKQSSSVSTFIVNFFFCLTTFLTIVE
Hydra ITLSMSLNLIICYVI LFILSISISDIVHT YVSVGASFLTYSISVSNIMQIVMISAIRVVALKR
Oikopleura MTIGVPANFLILYVS WLVINLALSDFLLL FLCKYYLTVKQLFFILSILIIATIAFDRYRMISS
Elegans LLYALISNMLILAGI WFIISIAICDILMT IRNIVMIFFYDLFWYTGVVQLGLMAGNRFVSIVY
Ascidia FCLSMAGNMFVLWS- RIMFYLALSDLIYT VMCRLCQMMKQFGMYASSFMVVVIGMDRVTAILS
Trichoplax GVLGSLANIAAIIVH YLLAHLAFADLIGT SFCAVLRFLNCVSTIASSSFAVLIAIERSKLILH
Nematostella AVVGTVSNLVVLVSF MFLISLLFADLFVC ENVTVRTFIGSLLLIASLNAIFCVMMDRFIAVQK
Drosophila VFGGVVGNSTLVLTL PLLLAVCIADLLVT VLCKVIHYIQVMPVAASTISFFMLSLDRYATVKH
Crassostrea FLCGFVLNSLTIHAI HLFLQLVALDFVAY ALCQISGALGTTCFICVFIFLTFLCAERMVKMNN
Branchiostoma GWFAFLGNILFIVRR FTFLNLHIADAIMG GLCQFTGFLFTLAAEIPFVALPVAVFEECIYAFY
Nigroviridis ATAIVGLNTSVWLCI MYMLSSCLSDICTG TFVSPTRTFHIVPTFLGLSFMAILAADRYHAVVS
Takifugu -VVGLVGNATLLRII ALIASLALGDLIYI CLCKLVPFLQKASVGITVLNLCALSVEQVQSRGV
Sarcophilus MTLGAVSNVLALAIL LFATGLVATDFAGV PACQFMGACMVFFGLCPLLLGCGMAVERCVGVTR
3PBL ILAIVFGNGLVCMAV YLVVSLAVADLLVA ICCDVFVTLDVMMCTASIWNLCAISIDRYTAVVM
4MBS FIFGFVGNMLVILIL IYLLNLAISDLFFL TMCQLLTGLYFIGFFSGIFFIILLTIDRYLAVVH
1.50
2.50
3.25
3.39
3.50
GCR1 LKVLNRWGYYPLILIGSWAFGTINRIHDFI FWLSVLDVGTAALMGLFNSIAYGF-
Amoeba ITYQFKLVNYIIVFLVCWIFAVVNRIINSL YTCNMLHTYLSISHGFYASLVFIY-
Entamoeba VSLVRKMAIYPFLYIVCWIVPFFNRLLPLF FWMEYVHCFTIPLLGFVNCLYYAN-
Paramecium EQYTKFLFYYTLIHFFCWIPILISSIFYII LWIGGISQIVACLQGFLDCLLFLI-
Hydra KAYLKVLLCSAIAYFVVWTPYSVVSLLSIF IF--TFCALFAKLSAVLNALVNCYI
Oikopleura QTLRRSIKLFTSVLVISWLPVVIWKIIR-L YGYSKFAETSLWIGPALNPILYSFL
Elegans MRLFIQFFVVSLVFLLTWTTWQWLPYMS-- KWAYFVMTSLFFINNSVNPTVYIIF
Ascidia RTILMR-TLITLTFLVCWGPYYGKGIYDWF APLDTAMYIVMYLNPVLHPIVFGV-
Trichoplax PWLIFFAIAIAITDATTWLPLSLAILLQPM KYVSISVILLQKVNMAIHPFIYS--
Nematostella TRCARAVRLVVGISLLCWLPTILVPVLIPI NRIWCYVLVMSSLNSCLDPFLYYWN
Drosophila RHLANMLIASALIFIVCWAPHVFCIFYKNF KSSVYFSLLLGYFYSAISPVIYWAL
Crassostrea FHLAATYVLILGIVFILWLPYVIVCYLDVY GGWYSIVLIISDISYCIKPIVFMSH
Branchiostoma HR----LFAVSFTNLSCWLPVVLFGMAGWD WAALIMIPI----NAAVNPLIYMIF
Nigroviridis RASVHLILVVAFLFLGAWLPLLFHVTVCNF NEGTDPFRILPRINAALTPVMYIRG
Takifugu REVAKAVFCLVLIFALCWFPLHLSILRKMG KRLDYLGMNLATINFCINPIILYFV
Sarcophilus AHDVEMVVGIMVVSCICWSPLLVLVVLAMG QRLLFLAVRLASWNQILDPWVYILL
3PBL KKATQMVAIVLGAFIVCWLPFFLTHVLNTH PELYSATTWLGYVNSALNPVIYTTF
4MBS HRDVRLIFTIMIVYFLFWAPYNIVLLLNTF DQAMQVTETLGMTHCCINPIIYAFV
6.48
7.45
7.49
7.53

Figure S9. Alignment of the helices 1-3 and 6-7 of the *Arabidopsis* GCR1 with cAMP receptor-type (class E) GPCRs from diverse eukaryotic groups and with two structures of class A GPCRs. The Taylor coloring scheme [36] was applied with 20% identity threshold. The positions that correspond to the Na^+ ligands in the human GPCR structures are highlighted.
The sequences are from the following organisms:
GCR1, *Arabidopsis thaliana* At1g48270 (UniProt: O04714);
Amoeba - *Dictyostelium aureostipes*, Amoebozoa (UniProt: B3VSF1_9MYCE);
Entamoeba - *Entamoeba histolytica*, Amoebozoa (UniProt: C4M112_ENTHI);
Paramecium - *Paramecium tetraurelia*, Ciliophora (UniProt: A0D441_PARTE);
Hydra - Hydra vulgaris, Cnidaria (UniProt: F1LIP2_HYDVU);
Oikopleura, *Oikopleura dioica*, Tunicata (UniProt: E4Y384_OIKDI);
Elegans - *Caenorhabditis elegans*, Nematoda (UniProt: Q17594_CAEEL);
Ascidia, *Ascidia intestinalis*, Tunicata (UniProt: Q869J1_CIOIN);
Trichoplax - *Trichoplax adhaerens*, Placozoa (UniProt: B3RU13_TRIAD);
Nematostella - *Nematostella vectensis*, sea anemone, Cnidaria (UniProt: A7RVZ2_NEMVE);
Drosophila - Drosophila persimilis, Fruit fly (UniProt: B4G6J2_DROPE);
Crassostrea, *Crassostrea gigas*, Pacific oyster (UniProt: K1PLP7_CRAGI);
Branchiostoma - *Branchiostoma floridae*, Florida lancelet (UniProt: C3YE30_BRAFL);
Nigroviridis - *Tetraodon nigroviridis*, Spotted green pufferfish (UniProt: Q4RUV4_TETNG);
Takifugu - *Takifugu rubripes*, Japanese pufferfish (UniProt: A3KE13_TAKRU);
Sarcophilus - *Sarcophilus harrisii*, Tasmanian devil (UniProt: G3VPS1_SARHA);
3PBL - human dopamine receptor (UniProt: P35462, DRD3_HUMAN) [21], and
4MBS - human CCR5 chemokine receptor (UniProt: P51681, CCR5_HUMAN) [27].

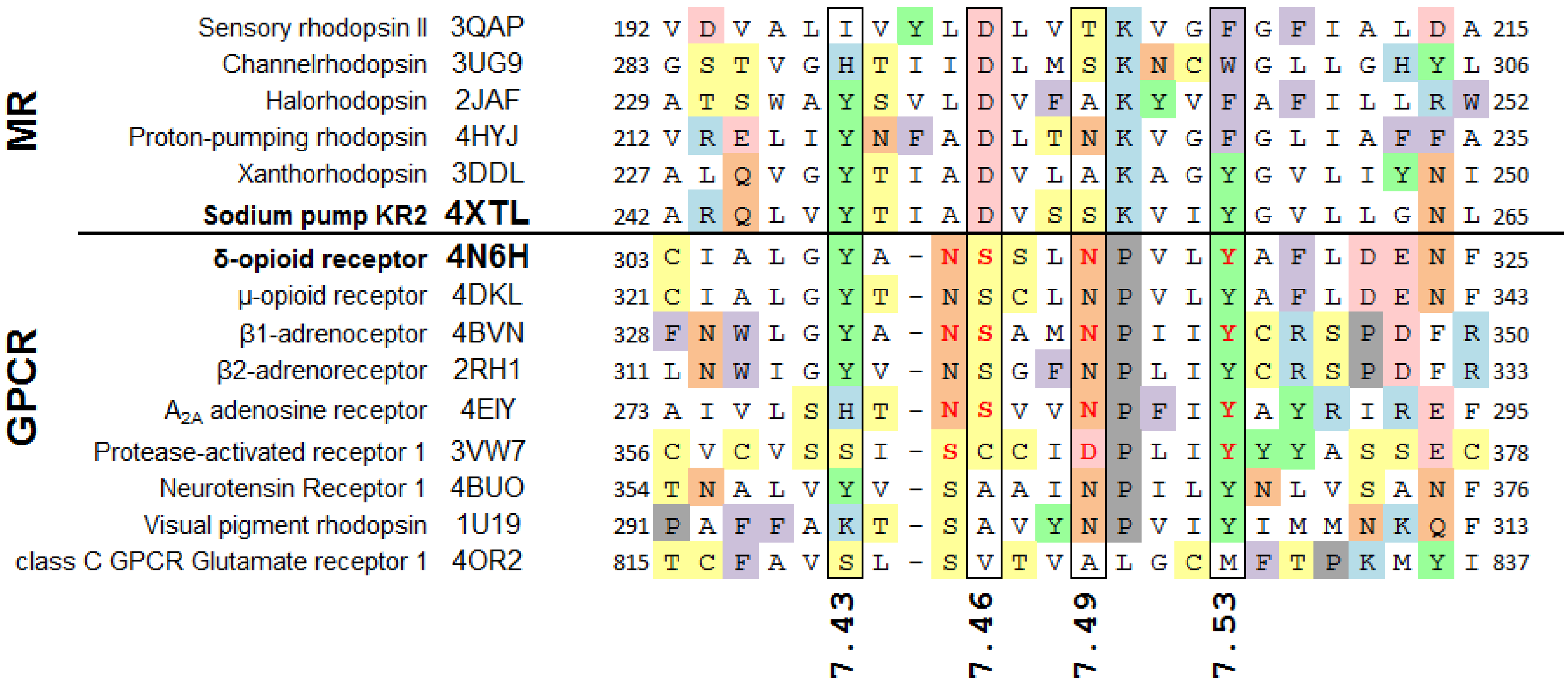

Figure S10a. Alternative multiple sequence alignment of helices G of MRs and helices 7 of GPCRs (see Table S1 for the list of all structures, Figure S10b for the structural explanation). The alignment is based on the superposition of KR2 and A_{2A} adenosine receptor (PDB 4EIY) structures (Fig. S3a,b). All the MR structures were aligned to the KR2 structure (PDB: 4XTL), and all the GPCR structures were aligned to A_{2A} adenosine receptor (PDB: 4EIY) using the results produced by the jFATCAT-rigid algorithm.

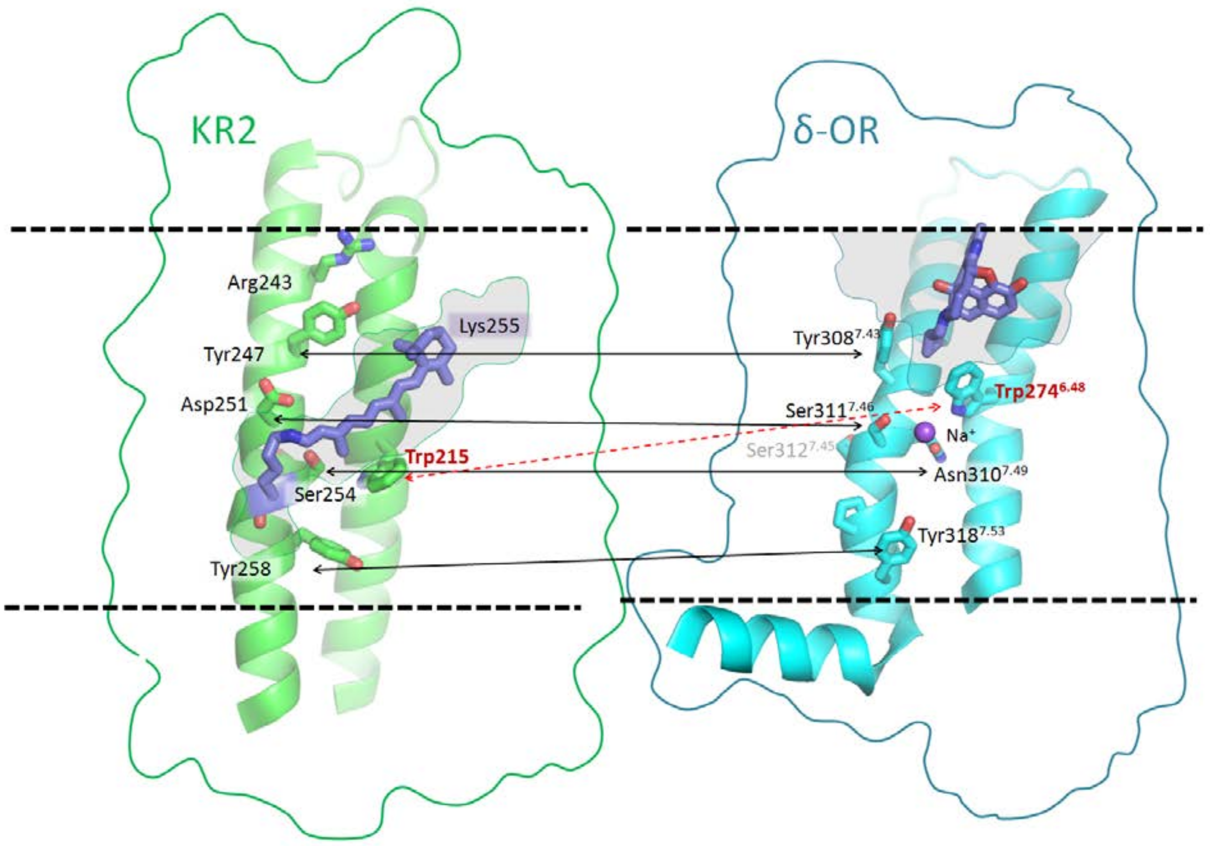

Figure S10b. Matching residues of helices G and F of KR2 (PDB: 4XTL) and helices 6 and 7 of δ-OR (PDB: 4N6H) according to the alternative alignment (Fig. S10a), see the main text and cf. with Figs. 1, S1-S4. The alternative alignment based on pairwise superposition shown in Fig. S3a,b provides a better match of putative Na^+ ligands in helices G and 7, but causes mismatch of conservative residues in all other helices, including functionally the conserved Trp residue in helices 6 and F, see Fig. S3. The KR2 structure is shown in green, the δ-OR structure is shown in blue. The Na^+ ion in δ-OR is shown as a purple sphere. The retinal molecule bound to the KR2 lysine residue and the signaling molecule in complex with δ-OR are shown as purple sticks. Sodium-binding residues marked by arrows match residues in brackets on Figure S10a.

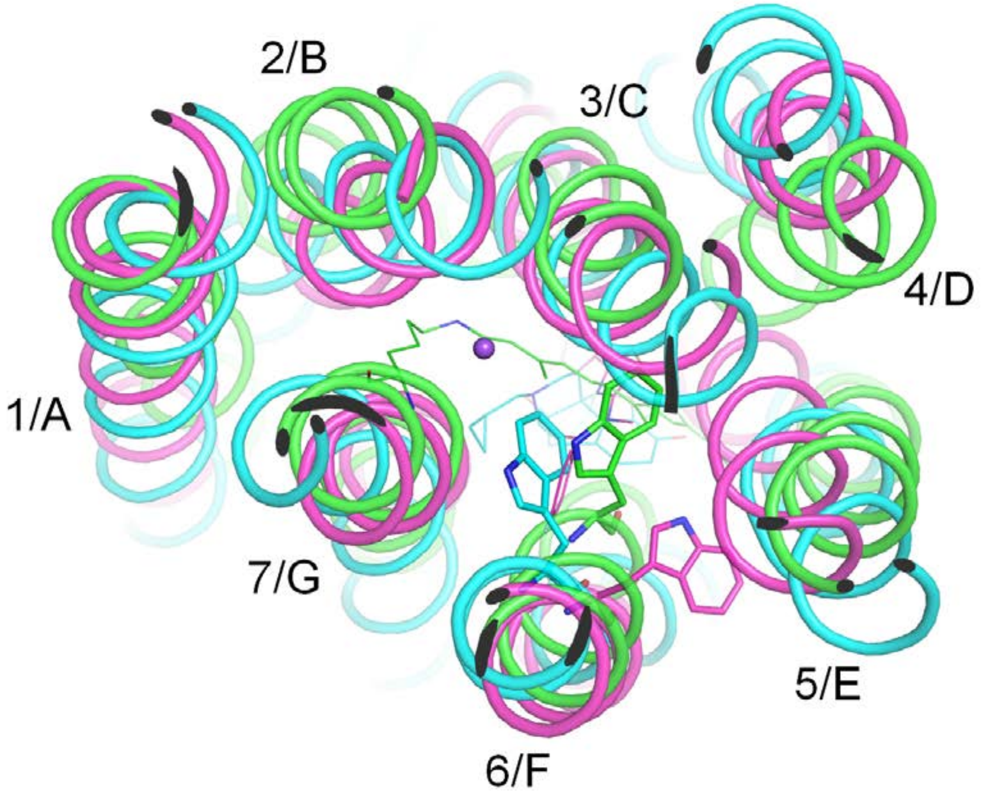

Figure S11a. Superposition of the class C human G protein-coupled metabotropic glutamate receptor 1 [PDB: 4OR2] (GluR, magenta) with δ-OR (blue) and KR2 (green). The PDBeFold server was used for the similarity search between the GluR and the whole PDB archive. Among the best matches were turkey β-1 adrenergic receptor [PDB: 2VT4] (RMSD 2.82 Å on 195 residues) and bacteriorhodopsin [PDB: 1X0K] (RMSD 3.87 Å on 189 residues). Those matches were used to build the presented superposition of GluR with δ-OR and KR2. The side chain of the conserved $Trp^{6.50}$ residue of GluR is turned towards the helix 5 and the movement of Trp aromatic ring seems to be hindered by the bound modulator molecule. Note that the Trp side chain in the MRs is in an intermediate position as compared to the class C and class A GPCRs.

```
4XTL IAYQFTSHILTLGYAVMLAGLLYFILTIKNV----D---KKF--QMSNILSAVVMVSAFLLLYAQAQNWT
4N6H SSLALAIAITALYSAVCAVGLLGNVLVMFGIVRYTK---MKTATNIYIFNLALADALATSTLPFQSAKYL
4OR2 YLEW-SNIESIIAIAFSCLGILVTLFVTLIFVLYRDTPVVKSSSRELCYIILAGIFLGYVCPFTLIAK--
                            |                              |
                            1.50                           2.50

4XTL SSFTFNEEVGRYFLDPSGDLFNNGYRYLNWLIDVPMLLFQI--LFVVSLT---------TS----KFSSV
4N6H METW-----------PFGELLCKAVLSIDY-YNMFTSIFTLTMMSVDRYIAVCHPVKALDFRTPAKAKLI
4OR2 ----------------PTTTSCYLQRLLVG-LSSAMC-YSALVTKTNRIARILARK---PRFMSAWAQVI
                                     |   |          |
                                     3.35           3.50
                                         3.39

4XTL -RNQ-FWFSGAMMIITGYIG------------------QFYEVSNLTAFLVWGAISSAFF-FHILWVMK
4N6H -NIC-IWVLASGVGVPIMVMAVTR------PRDGAVVCMLQFPSPSWYWDTVTKICVFLFAFVVPILIIT
4OR2 IASILIS-VQLTLVVTLIIMEPPMPILSYPSIKEVYLICNT--------SNLGVVAPLGYNGLLIMSCTY
           |                                                         |
           4.50                                                      5.50

4XTL KVINEGKEG---------------ISP--AGQKILSNIWILFLISWTLYPG--AYLMPYLTGVDGFLYSE
4N6H VCYGLMLLRLRSVRLLSGSKEKDRSLR--RITRMVLVVVGAFVVCWAPIHI--FVIVWTLVD--IDRRDP
4OR2 YAFKTRN-VP----------------ANFNEAKYIAFTMYTTCIIWLAFVPIYF--GS-----------
                                                  | |
                                               6.48 6.50

4XTL DGVMARQLVYTIADVSSKVIYGVL-LGNLAI--TLS
4N6H LVVAALHLCIALGYA-NSSLNPVL-YAFLD--ENFK
4OR2 --NYKIITTCFAVSL-SVTVALGCMFT-PKMYIII-
                     |   ||   |
                  7.45    7.50 7.53
                         7.49
```

Figure S11b. Sequence alignment based on the structure superposition of KR2 (PDB: 4XTL), δ-OR (PDB: 4N6H) and class C GPCR glutamate receptor 1 (hereafter GluR) [PDB: 4OR2].

Structural superposition is shown on Figure S11a. Sequence alignment was derived from PDBeFold server [3] results. The Na$^+$-binding residues are shown in red, the reference 50th residues for the Ballesteros-Weinstein numbering scheme [31, 32] are shown in bold font. Notably, canonical alignment of GPCR sequences[32, 40] presumes superposition of proline residues in helix 6, with Pro$^{\mathbf{6.50}}$ being the reference residue for the numbering scheme, which leads to the alignment of Trp$^{\mathbf{6.48}}$ in class A GPCRs with a phenylalanine residue in GluR. However, our structure-based alignment matched tryptophan residues in position 6.48 (numbering by δ-OR), leading to a mismatch of proline residues downstream.

References

1. Gushchin I, Shevchenko V, Polovinkin V, Kovalev K, Alekseev A, Round E, Borshchevskiy V, Balandin T, Popov A, Gensch T *et al*: **Crystal structure of a light-driven sodium pump**. *Nat Struct Mol Biol* 2015, **22**(5):390-395.
2. Fenalti G, Giguere PM, Katritch V, Huang XP, Thompson AA, Cherezov V, Roth BL, Stevens RC: **Molecular control of delta-opioid receptor signalling**. *Nature* 2014, **506**(7487):191-196.
3. Krissinel E, Henrick K: **Secondary-structure matching (SSM), a new tool for fast protein structure alignment in three dimensions**. *Acta Crystallogr D Biol Crystallogr* 2004, **60**(Pt 12 Pt 1):2256-2268.
4. Rose PW, Bi CX, Bluhm WF, Christie CH, Dimitropoulos D, Dutta S, Green RK, Goodsell DS, Prlic A, Quesada M *et al*: **The RCSB Protein Data Bank: new resources for research and education**. *Nucleic Acids Res* 2013, **41**(D1):D475-D482.
5. Ye YZ, Godzik A: **Flexible structure alignment by chaining aligned fragment pairs allowing twists**. *Bioinformatics* 2003, **19**:Ii246-Ii255.
6. Prlic A, Bliven S, Rose PW, Bluhm WF, Bizon C, Godzik A, Bourne PE: **Pre-calculated protein structure alignments at the RCSB PDB website**. *Bioinformatics* 2010, **26**(23):2983-2985.
7. Zhang C, Srinivasan Y, Arlow DH, Fung JJ, Palmer D, Zheng Y, Green HF, Pandey A, Dror RO, Shaw DE *et al*: **High-resolution crystal structure of human protease-activated receptor 1**. *Nature* 2012, **492**(7429):387-392.
8. Liu W, Chun E, Thompson AA, Chubukov P, Xu F, Katritch V, Han GW, Roth CB, Heitman LH, IJzerman AP *et al*: **Structural basis for allosteric regulation of GPCRs by sodium ions**. *Science* 2012, **337**(6091):232-236.
9. Miller-Gallacher JL, Nehme R, Warne T, Edwards PC, Schertler GF, Leslie AG, Tate CG: **The 2.1 Å resolution structure of cyanopindolol-bound beta1-adrenoceptor identifies an intramembrane Na^+ ion that stabilises the ligand-free receptor**. *PLoS One* 2014, **9**(3):e92727.
10. Katritch V, Fenalti G, Abola EE, Roth BL, Cherezov V, Stevens RC: **Allosteric sodium in class A GPCR signaling**. *Trends Biochem Sci* 2014, **39**(5):233-244.
11. Schrodinger, LLC: **The PyMOL Molecular Graphics System, Version 1.3r1**. 2010.
12. Kanada S, Takeguchi Y, Murakami M, Ihara K, Kouyama T: **Crystal structures of an O-like blue form and an anion-free yellow form of *Pharaonis* halorhodopsin**. *J Mol Biol* 2011, **413**(1):162-176.
13. Gushchin I, Reshetnyak A, Borshchevskiy V, Ishchenko A, Round E, Grudinin S, Engelhard M, Buldt G, Gordeliy V: **Active State of sensory rhodopsin II: structural determinants for signal transfer and proton pumping**. *J Mol Biol* 2011, **412**(4):591-600.
14. Kato HE, Zhang F, Yizhar O, Ramakrishnan C, Nishizawa T, Hirata K, Ito J, Aita Y, Tsukazaki T, Hayashi S *et al*: **Crystal structure of the channelrhodopsin light-gated cation channel**. *Nature* 2012, **482**(7385):369-374.
15. Gmelin W, Zeth K, Efremov R, Heberle J, Tittor J, Oesterhelt D: **The crystal structure of the L1 intermediate of halorhodopsin at 1.9 Å resolution**. *Photochem Photobiol* 2007, **83**(2):369-377.

16. Ran TT, Ozorowski G, Gao YY, Sineshchekov OA, Wang WW, Spudich JL, Luecke H: **Cross-protomer interaction with the photoactive site in oligomeric proteorhodopsin complexes**. *Acta Crystallogr D* 2013, **69**:1965-1980.
17. Gushchin I, Chervakov P, Kuzmichev P, Popov AN, Round E, Borshchevskiy V, Ishchenko A, Petrovskaya L, Chupin V, Dolgikh DA *et al*: **Structural insights into the proton pumping by unusual proteorhodopsin from nonmarine bacteria**. *Proc Natl Acad Sci USA* 2013, **110**(31):12631-12636.
18. Luecke H, Schobert B, Stagno J, Imasheva ES, Wang JM, Balashov SP, Lanyi JK: **Crystallographic structure of xanthorhodopsin, the light-driven proton pump with a dual chromophore**. *Proc Natl Acad Sci USA* 2008, **105**(43):16561-16565.
19. Manglik A, Kruse AC, Kobilka TS, Thian FS, Mathiesen JM, Sunahara RK, Pardo L, Weis WI, Kobilka BK, Granier S: **Crystal structure of the μ-opioid receptor bound to a morphinan antagonist**. *Nature* 2012, **485**(7398):321-326.
20. Cherezov V, Rosenbaum DM, Hanson MA, Rasmussen SGF, Thian FS, Kobilka TS, Choi HJ, Kuhn P, Weis WI, Kobilka BK *et al*: **High-resolution crystal structure of an engineered human beta(2)-adrenergic G protein-coupled receptor**. *Science* 2007, **318**(5854):1258-1265.
21. Chien EYT, Liu W, Zhao QA, Katritch V, Han GW, Hanson MA, Shi L, Newman AH, Javitch JA, Cherezov V *et al*: **Structure of the human dopamine D3 receptor in complex with a D2/D3 selective antagonist**. *Science* 2010, **330**(6007):1091-1095.
22. Zhang C, Srinivasan Y, Arlow DH, Fung JJ, Palmer D, Zheng YW, Green HF, Pandey A, Dror RO, Shaw DE *et al*: **High-resolution crystal structure of human protease-activated receptor 1**. *Nature* 2012, **492**(7429):387-392.
23. Egloff P, Hillenbrand M, Klenk C, Batyuk A, Heine P, Balada S, Schlinkmann KM, Scott DJ, Schutz M, Pluckthun A: **Structure of signaling-competent neurotensin receptor 1 obtained by directed evolution in *Escherichia coli***. *Proc Natl Acad Sci U S A* 2014, **111**(6):E655-662.
24. Wang C, Jiang Y, Ma JM, Wu HX, Wacker D, Katritch V, Han GW, Liu W, Huang XP, Vardy E *et al*: **Structural basis for molecular recognition at serotonin receptors**. *Science* 2013, **340**(6132):610-614.
25. Hanson MA, Roth CB, Jo EJ, Griffith MT, Scott FL, Reinhart G, Desale H, Clemons B, Cahalan SM, Schuerer SC *et al*: **Crystal structure of a lipid G protein-coupled receptor**. *Science* 2012, **335**(6070):851-855.
26. Kruse AC, Ring AM, Manglik A, Hu JX, Hu K, Eitel K, Hubner H, Pardon E, Valant C, Sexton PM *et al*: **Activation and allosteric modulation of a muscarinic acetylcholine receptor**. *Nature* 2013, **504**(7478):101-+.
27. Tan Q, Zhu Y, Li J, Chen Z, Han GW, Kufareva I, Li T, Ma L, Fenalti G, Zhang W *et al*: **Structure of the CCR5 chemokine receptor-HIV entry inhibitor maraviroc complex**. *Science* 2013, **341**(6152):1387-1390.
28. Okada T, Sugihara M, Bondar AN, Elstner M, Entel P, Buss V: **The retinal conformation and its environment in rhodopsin in light of a new 2.2 Å crystal structure**. *J Mol Biol* 2004, **342**(2):571-583.
29. Wu HX, Wang C, Gregory KJ, Han GW, Cho HP, Xia Y, Niswender CM, Katritch V, Meiler J, Cherezov V *et al*: **Structure of a class C GPCR metabotropic glutamate receptor 1 bound to an allosteric modulator**. *Science* 2014, **344**(6179):58-64.

30. Krissinel E, Henrick K: **Secondary-structure matching (SSM), a new tool for fast protein structure alignment in three dimensions**. *Acta Crystallogr D* 2004, **60**:2256-2268.
31. Ballestros JA, Weinstein H: **Integrated methods for the construction of three-dimensional models and computational probing of structure-function relations in G protein-coupled receptors**. *Methods Neurosci* 1995, **25**:366-428.
32. Isberg V, de Graaf C, Bortolato A, Cherezov V, Katritch V, Marshall FH, Mordalski S, Pin JP, Stevens RC, Vriend G *et al*: **Generic GPCR residue numbers - aligning topology maps while minding the gaps**. *Trends Pharmacol Sci* 2015, **36**(1):22-31.
33. Holm L, Rosenstrom P: **Dali server: conservation mapping in 3D**. *Nucleic Acids Res* 2010, **38**:W545-W549.
34. Kato HE, Inoue K, Abe-Yoshizumi R, Kato Y, Ono H, Konno M, Hososhima S, Ishizuka T, Hoque MR, Kunitomo H *et al*: **Structural basis for Na^+ transport mechanism by a light-driven Na^+ pump**. *Nature* 2015, **521**(7550):48-53.
35. Waterhouse AM, Procter JB, Martin DMA, Clamp M, Barton GJ: **Jalview Version 2-a multiple sequence alignment editor and analysis workbench**. *Bioinformatics* 2009, **25**(9):1189-1191.
36. Taylor WR: **The classification of amino acid conservation**. *J Theor Biol* 1986, **119**(2):205-218.
37. Nakanishi T, Kanada S, Murakami M, Ihara K, Kouyama T: **Large deformation of helix F during the photoreaction cycle of *Pharaonis* halorhodopsin in complex with azide**. *Biophys J* 2013, **104**(2):377-385.
38. Haga K, Kruse AC, Asada H, Yurugi-Kobayashi T, Shiroishi M, Zhang C, Weis WI, Okada T, Kobilka BK, Haga T *et al*: **Structure of the human M2 muscarinic acetylcholine receptor bound to an antagonist**. *Nature* 2012, **482**(7386):547-U147.
39. Inoue K, Ono H, Abe-Yoshizumi R, Yoshizawa S, Ito H, Kogure K, Kandori H: **A light-driven sodium ion pump in marine bacteria**. *Nat Commun* 2013, **4**:1678.
40. Isberg V, Vroling B, van der Kant R, Li K, Vriend G, Gloriam D: **GPCRDB: an information system for G protein-coupled receptors**. *Nucleic Acids Res* 2014, **42**(Database issue):D422-D425.
